# New insights into predator–prey dynamics: First evidence of a leopard cat hunting coypus

**DOI:** 10.1002/ece3.11016

**Published:** 2024-02-13

**Authors:** Hee‐Bok Park, Anya Lim

**Affiliations:** ^1^ Research Center for Endangered Species National Institute of Ecology Yeongyang Gyeongbuk Korea

**Keywords:** cooperative defense, coypu, interspecies interaction, invasive prey, leopard cat

## Abstract

We present the first documented evidence of interactions between the leopard cat (*Prionailurus bengalensis euptilurus*) and the invasive coypu (*Myocastor coypus*) in South Korea, captured through camera traps in Hwapocheon wetland park from May 2015 to April 2017. Two interactions were recorded: one showing a leopard cat carrying a carcass of sub‐adult coypu and the other a 4‐min sequence of predation and defense between two species. The observed interactions indicate active predatory behavior by the leopard cat against coypus and cooperative defense by coypus. These findings shed new light on predator–prey dynamics, highlighting the leopard cat's potential role as a predator of coypus and coypus' defensive abilities. Understanding these relationships could facilitate more effective management of invasive species and offer broader implications for ecosystem dynamics and conservation strategies.

## Graphical abstract

This study provides the first documented evidence of interactions between the native leopard cat and the invasive coypu in South Korea, observed in the Hwapocheon wetland park from May 2015 to April 2017. Through camera traps, the research unveils active predatory behavior by leopard cats against coypus and the cooperative defense mechanisms exhibited by the coypus.

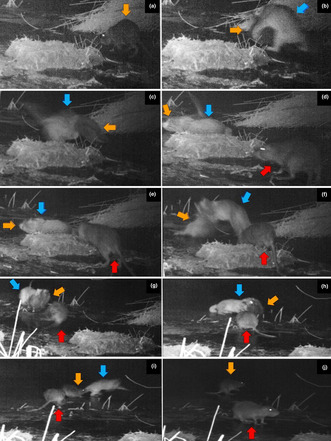

The leopard cat (*Prionailurus bengalensis*), designated as Least Concern (LC) by the IUCN Redlist, is a prevalent species residing in diverse habitats, such as forests, agricultural areas, and riparian regions (Lorica & Heaney, 2013; Mohamed et al., 2013; Nakanishi & Izawa, 2016; Rajaratnam et al., 2007; Ross et al., 2015). As obligate carnivores, they demonstrate a wide range of hunting abilities, primarily targeting small rodents and showcasing agility in swimming and climbing (Chua et al., 2016; Lee et al., 2014; Nakanishi & Izawa, 2016; Rajaratnam et al., 2007; Seryodkin & Burkovskiy, 2019; Shehzad et al., 2012). Their prey preferences vary regionally. For example, Japanese Iriomote cats (*P. b. iriomotensis*) focus on birds and amphibians, whereas in Korea, leopard cats (*P. b. euptilurus)* predominantly consume small rodents (Lee et al., 2014; Nakanishi & Izawa, 2016). Specifically in South Korea, leopard cats serve as the only extant *Felidae* species, a top predator, and a protected species, weighing between 3 and 7 kg (Jo et al., 2018).

Contrastingly, the coypu (*Myocastor coypus*), a semi‐aquatic rodent weighing between 5 and 9 kg, ranks among the IUCN World's Top 100 Invasive Alien Species due to its detrimental impact on aquatic ecosystems, crops, and drainage systems (Adhikari et al., 2022; Kim, Choi, et al., 2019; Lowe et al., 2000). Since its introduction to South Korea in 1985 to augment farm income, escaped coypus have proliferated along the Nakdong River, causing noticeable damage to wetlands and crops through their insatiable appetite, high reproductive capacity, and burrowing behavior (Hong et al., 2015). Attempts to manage their population have included bounty hunting since 2014 (Adhikari et al., 2022; Kim, Lim, et al., 2019). Although predators such as alligators *(Allidator mississippiensis*), coyotes (*Canis latrans)*, pumas (*Puma concolor*), and ocelots *(Leopardus pardalis*) consume coypus in the Americas (Burnam & Mengak, 2007; Gabrey et al., 2009; Mcvey, 2012; Wolfe et al., 1987), their interactions with native predators in Korea are largely unknown. While leopard cats and Eurasian otters (*Lutra lutra*) are presumed potential predators of coypus, no direct evidence of leopard cats preying on coypus has been documented previously in Korea or elsewhere. We present herein the first observed evidence of this hitherto unexplored interaction.

Between May 2015 to April 2017, 16 camera traps (still‐photograph movement triggered model: Hyperfire HC550 and HC600; Reconyx, Inc USA) were deployed at Hwapocheon wetland park (1.59 km^2^) in Gimhae, a major habitat for coypus in South Korea to study leopard cat habitat. We set camera traps to take three consecutive photos without intervals when motion is detected. Of these cameras, two captured leopard cat–coypu interactions. The initial occurrence, recorded on November 16, 2015, at 18:05, displayed a leopard cat carrying a sub‐adult coypu (Figure 1). Both the leopard cat and coypu were wet, leading us to assume that the leopard cat might have hunted the sub‐adult coypu near the water edge. However, this single observation does not conclusively demonstrate predation, and we cannot rule out the possibility that the leopard cat was carrying the coypu carcass as bycatch, rather than having actively hunted it. The subsequent direct record of predation, on January 16, 2017, at 23:30, portrayed a 4‐min sequence of predatory and defensive behaviors between a leopard cat and two coypus, providing clearer evidence of predatory behavior (Figure 2, Table 1, Video S1).

**FIGURE 1:**
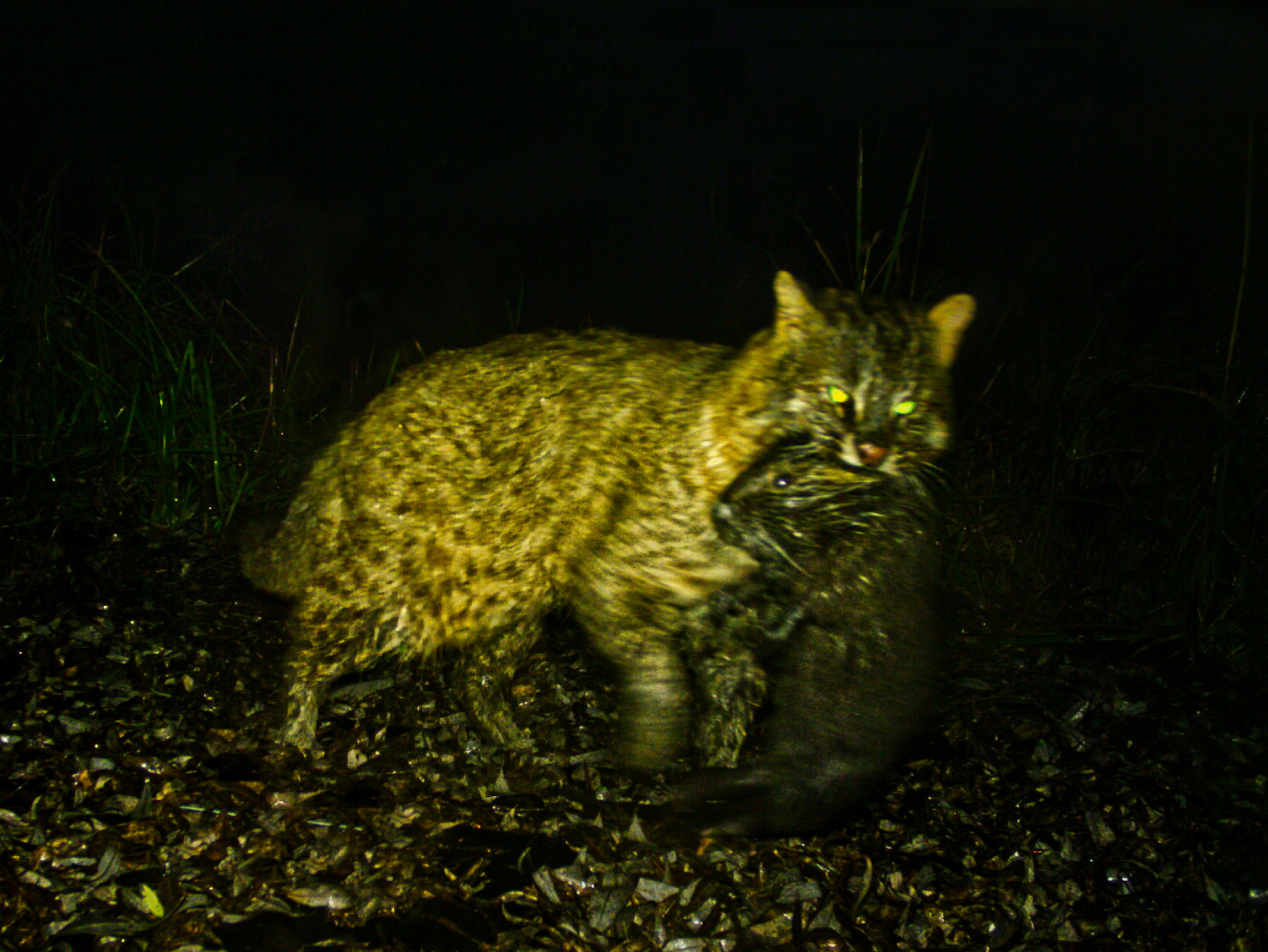
The first recorded prey–predator interaction between a leopard cat (*Prionailurus bengalensis euptilurus*) and a coypu (*Myocastor coypus*), captured on November 16, 2015, at 18:05. Adapted from (Lee et al., 2015).

**FIGURE 2:**
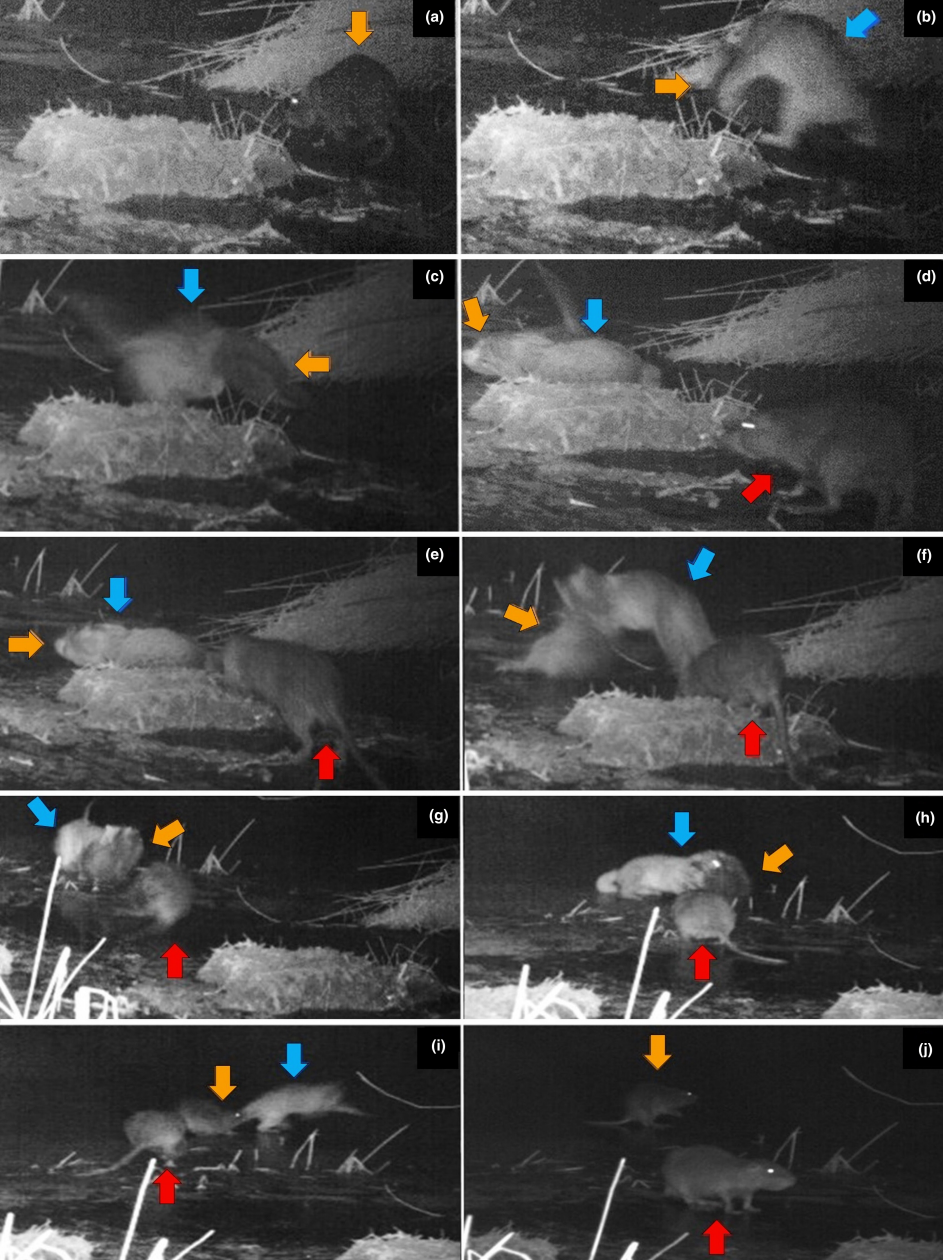
Selected photo sequence illustrating the predatory and defensive behaviors of a leopard cat (indicated by a blue arrow) and coypus (indicated by orange and red arrows). The sequence was recorded on January 16, 2017, from 23:30:46 to 23:34:40 and progresses from top left (a) to bottom right (j): (a) appearance of a coypu; (b–i) leopard cat engaging in hunting behavior and coypus in defensive actions; and (j) coypus patrolling the perimeter.

**TABLE 1:** Chronology of photo‐documented interactions between the leopard cat (*Prionailurus bengalensis euptilurus*) and the invasive coypus (*Myocastor coypus*) in the Hwapocheon wetland park, South Korea on January 16, 2017.

Time of day	Activity description
23:30:46	An adult coypu moves through a frozen wetland. Figure 1a
23:30:46	A leopard cat initiates predation on the coypu, climbing onto its back and grasping it with its claws. Figure 1b
23:30:47	Coypu resists; the leopard cat changes position. Figure 1c
23:30:56	First coypu is subdued; a second coypu emerges. Figure 1d
23:30:57	Second coypu moves toward the rear of the leopard cat. Figure 1e
23:30:58	Leopard cat jumps while biting the coypu; second coypu continues approaching. Figure 1f
23:30:59	Leopard cat changes position again while biting the coypu; second coypu continues approaching. Figure 1g
23:30:59	Leopard cat is still biting coypu's neck; second coypu is nearby. Figure 1h
23:31:00	Leopard cat releases bitten coypus and moves away. Figure 1i
23:31:01–23:32:08	Coypus appear alert, looking in the direction the leopard cat moved. Figure 1j
23:32:49	Alertness continues in coypus, followed by movement. Video S1
23:32:50	Coypus move. Video S1
23:33:48	Leopard cat reappears. Video S1
23:33:49–23:33:50	Leopard cat looks around. Video S1
23:34:02	Coypu reappears and chases; leopard cat runs. Video S1
23:34:03–23:34:06	Coypu continue chasing in the direction of the leopard cat. Video S1
23:34:40	Coypu returns. Video S1

In weather conditions measuring −6°C, an adult coypu was recorded traversing the frozen surface of the Hwapocheon wetland (Figure 2a). Subsequently, a leopard cat was observed approaching the coypu, climbing onto its back and grasping it with its claws. Then, the leopard cat bit the coypu's neck and subdued it (Figure 2b,c). During this predatory behavior, a second coypu appeared (Figure 2d), approaching the leopard cat from behind. This caused the leopard cat to change its position, although it continued biting the first coypu (Figure 2e,f). The images further depict the second coypu approaching the leopard cat in what can be described as a threatening manner, resulting in a standoff (Figure 2g,h). The subsequent defensive behavior exhibited by the coypus caused the leopard cat to flee the scene (Figure 2i). The coypus were then observed patrolling the area in the direction of the leopard cat's escape (Figure 2j), with their vigilance lasting for 1 min and 7 s before exiting the camera frame (Table 1; Video S1). Later, the leopard cat reappeared, seemingly on guard in the area. The sequence concluded with two coypus emerging and charging at the leopard cat, causing it to flee once more.

Among 207 scholarly papers, indexed on Google Scholar, related to the leopard cat published between 1952 and 2022, no instances of coypu predation were reported. Our observation revealed three of the four stages of felid feeding behavior, smell/hearing—stalking—hunting—killing the prey, in relation to coypus (Lindburg, 1988; Resende et al., 2009). The leopard cat was unable to complete the killing stage due to the defensive actions of a second coypu. Hunting of large prey, equivalent in size to adult coypus, is less common for leopard cats than targeting small rodents, but given instances of predation on large avian species, such as red‐crowned crane (*Grus japonensis*) and whooper swan (*Cygnus cygnus*), the success of individual leopard cats in hunting large prey appears plausible (Lee, 2012).

These observations signify that the leopard cat engages in predation of the invasive coypu in South Korea, though no native predator of predation for coypus was known previously. Particularly noteworthy was the recording of a leopard cat's hunting process of an adult coypu, substantiating the possibility of leopard cats as coypu predators. We also confirmed the coypus' ability to collectively defend against carnivores. These findings offer novel insights into the predator–prey dynamics between these species, enriching our understanding of leopard cats' ecological roles, especially in regions impacted by invasive species like the coypu.

Future research must delve further into predator–prey interactions between native predators and invasive species, exploring their effects on coypu populations. Understanding these relationships could facilitate more effective management of invasive species and offer broader implications for ecosystem dynamics and conservation strategies.

## AUTHOR CONTRIBUTIONS

**Hee‐Bok Park:** Conceptualization (lead); writing – original draft (supporting); writing – review and editing (supporting). **Anya Lim:** Conceptualization (supporting); writing – original draft (lead); writing – review and editing (lead).

## CONFLICT OF INTEREST STATEMENT

The authors declare that there are no conflicts of interest.

## Supporting information


Data S1.

Video S1.

## Data Availability

The data supporting the findings of this study are available within this article (Figures 1 and 2). All captured photos along with a video created from these images, are available in the Supporting Information.
