# Supplementary figures and images for: New insights into predator–prey dynamics: First evidence of a leopard cat hunting coypus
Source: Ecol Evol. 2024 Feb 13;14(2):e11016. doi: 10.1002/ece3.11016 (PMC10862177; doi:10.1002/ece3.11016)

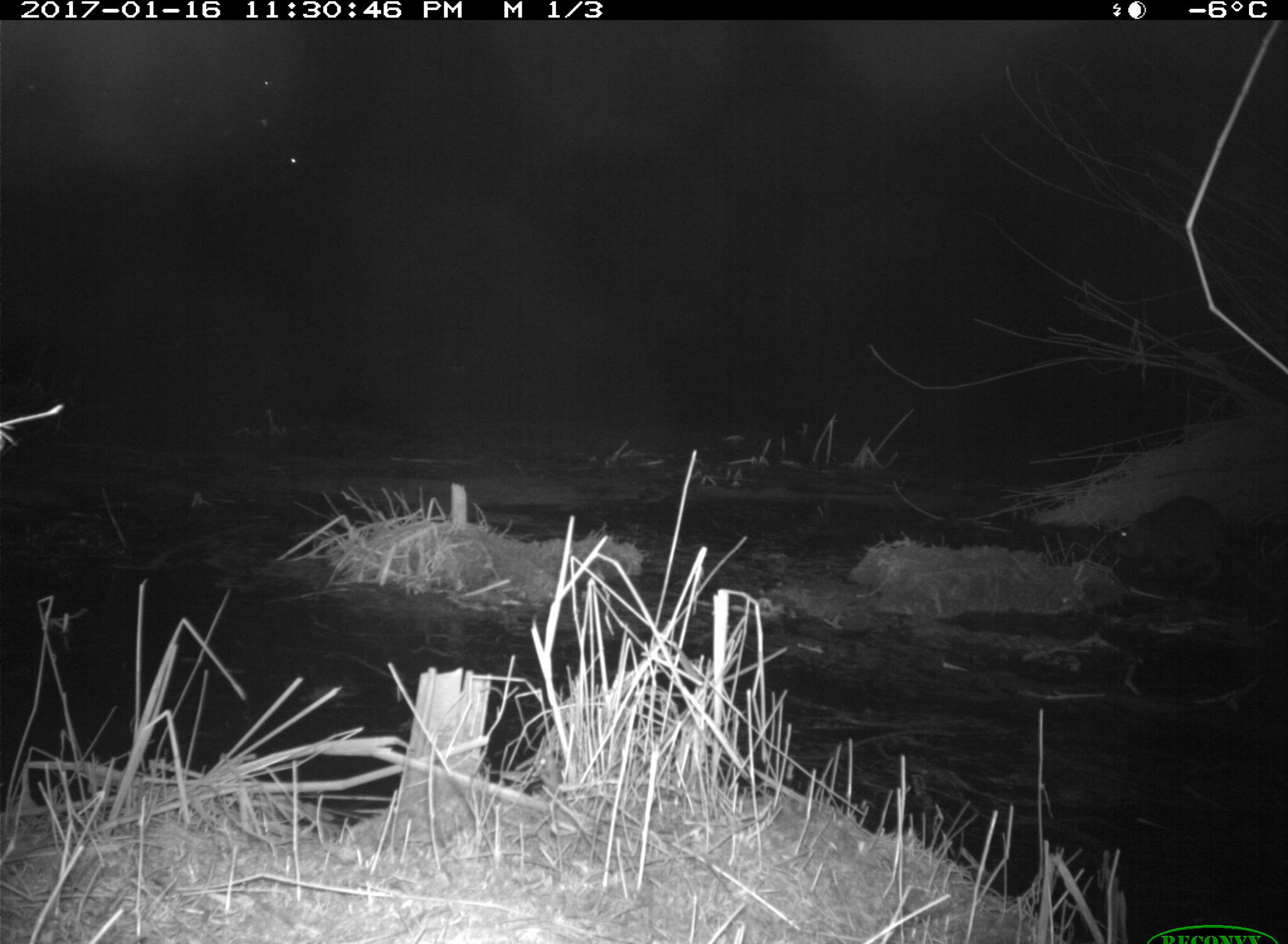

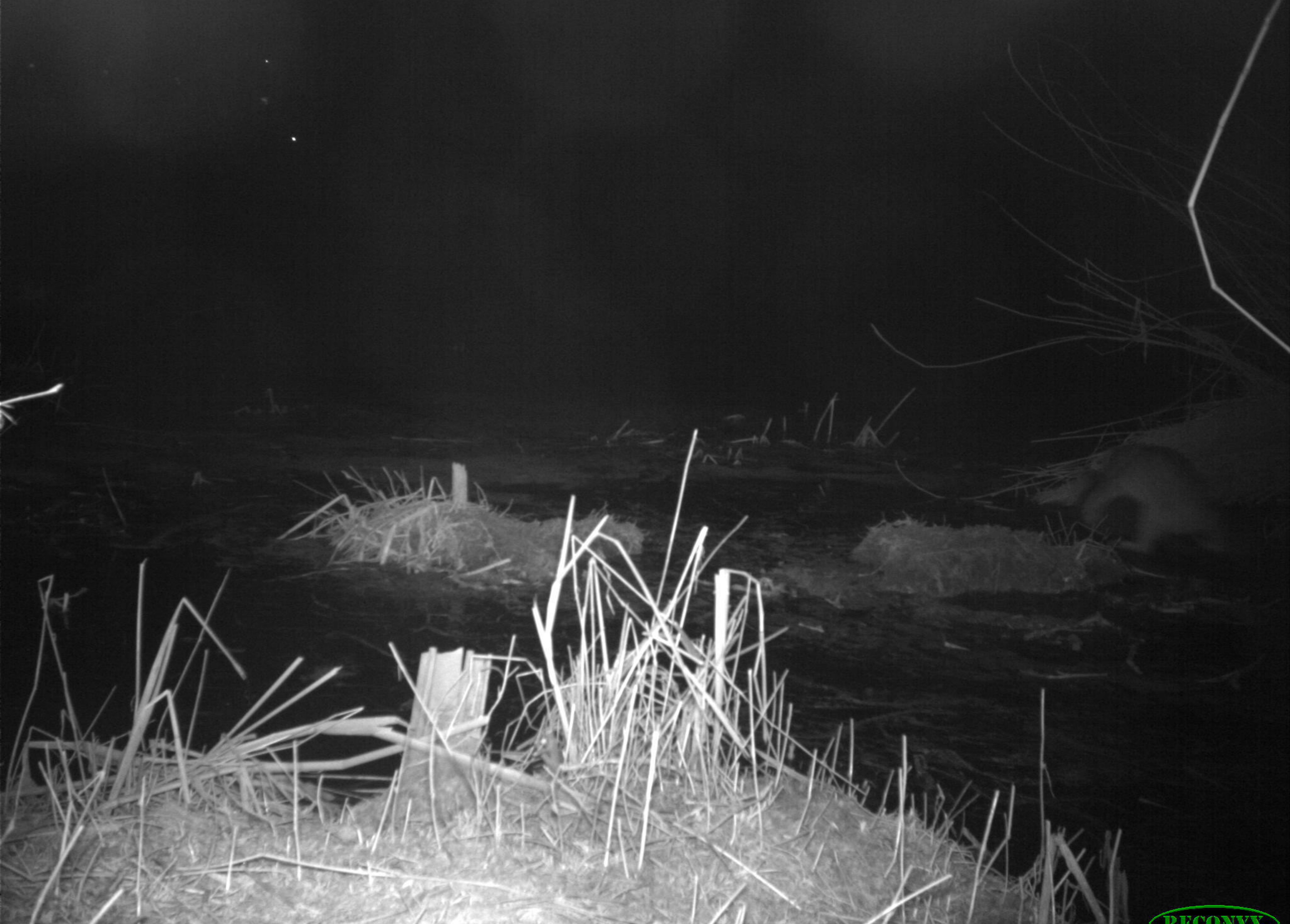

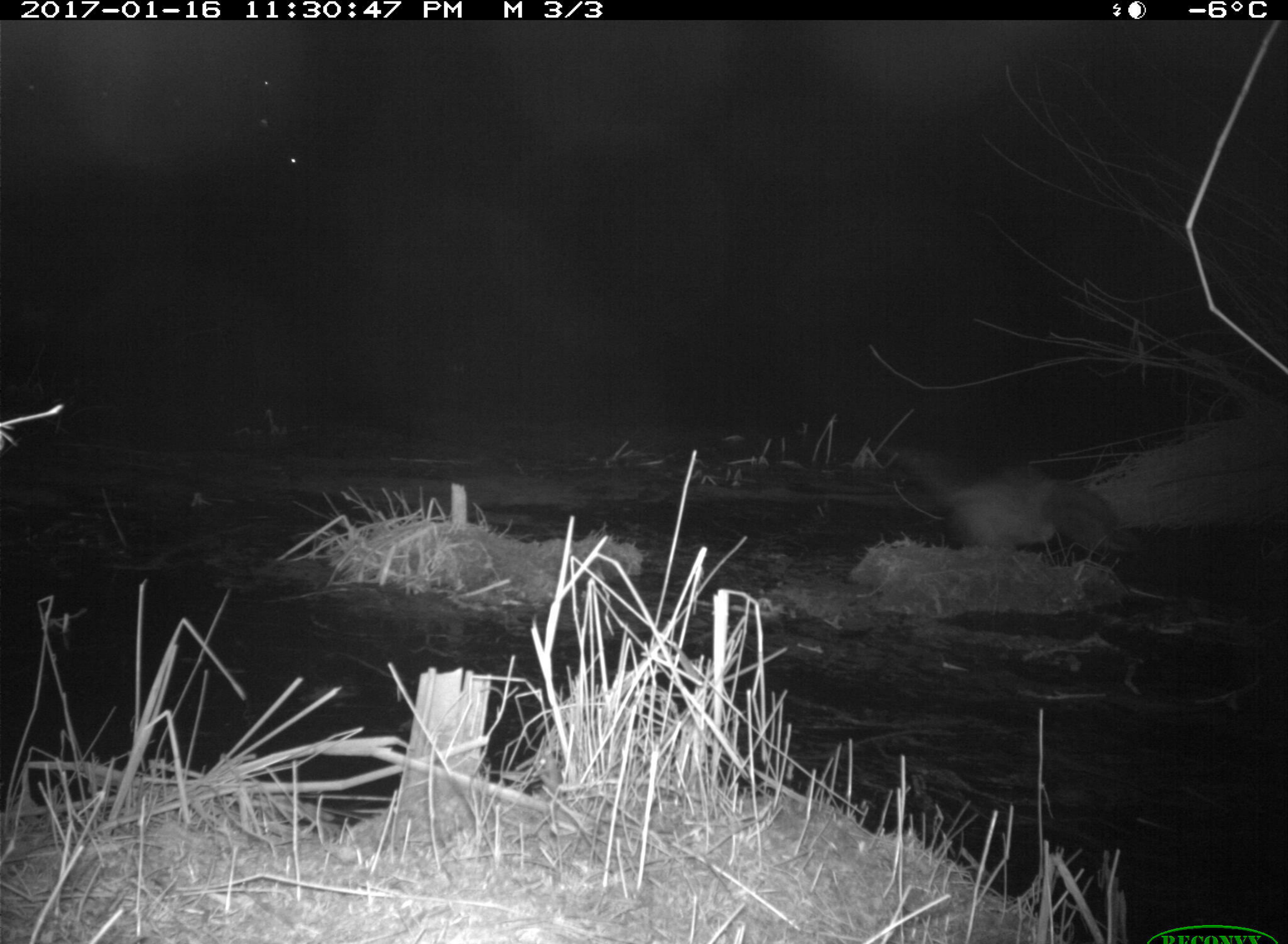

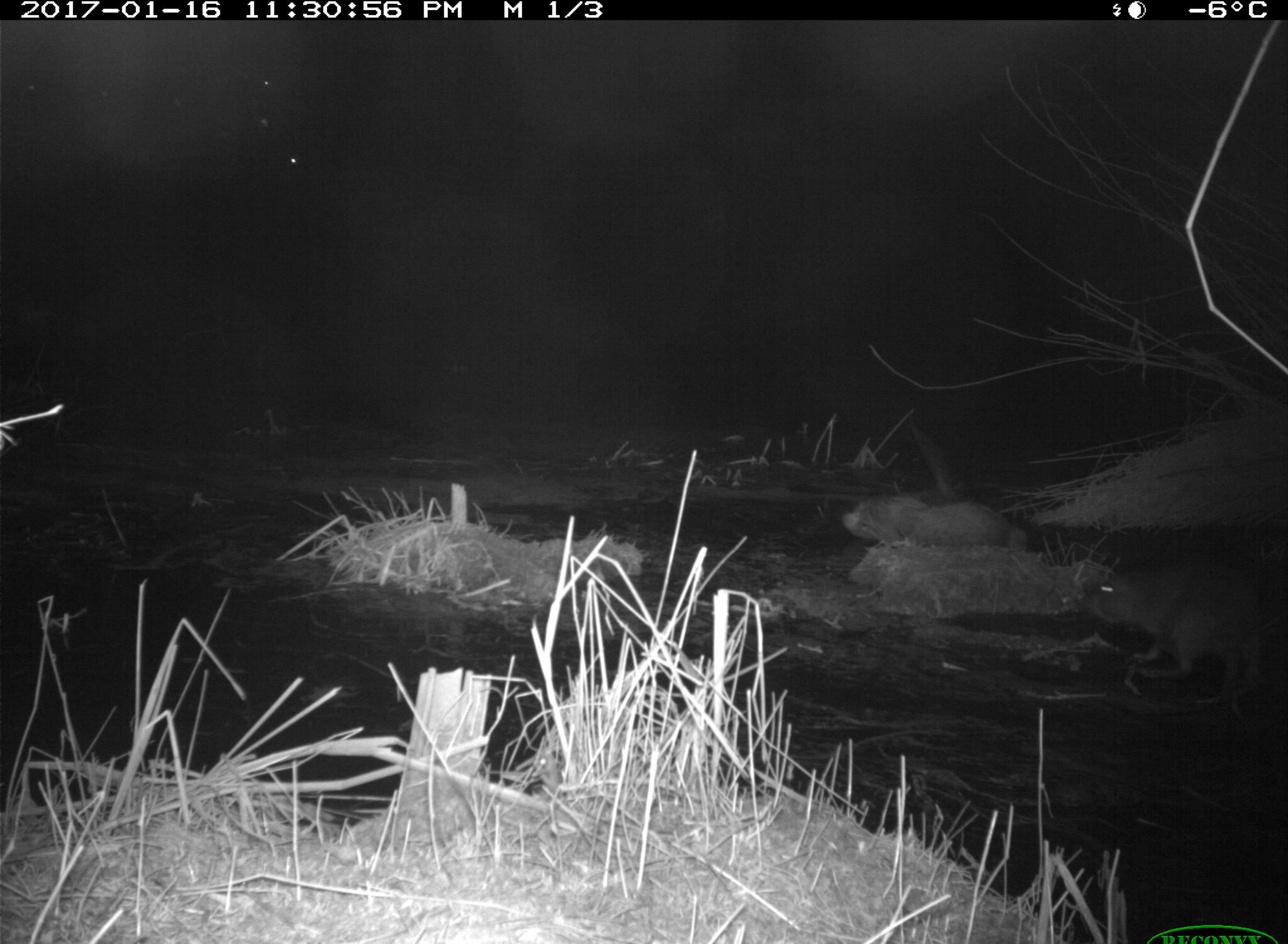

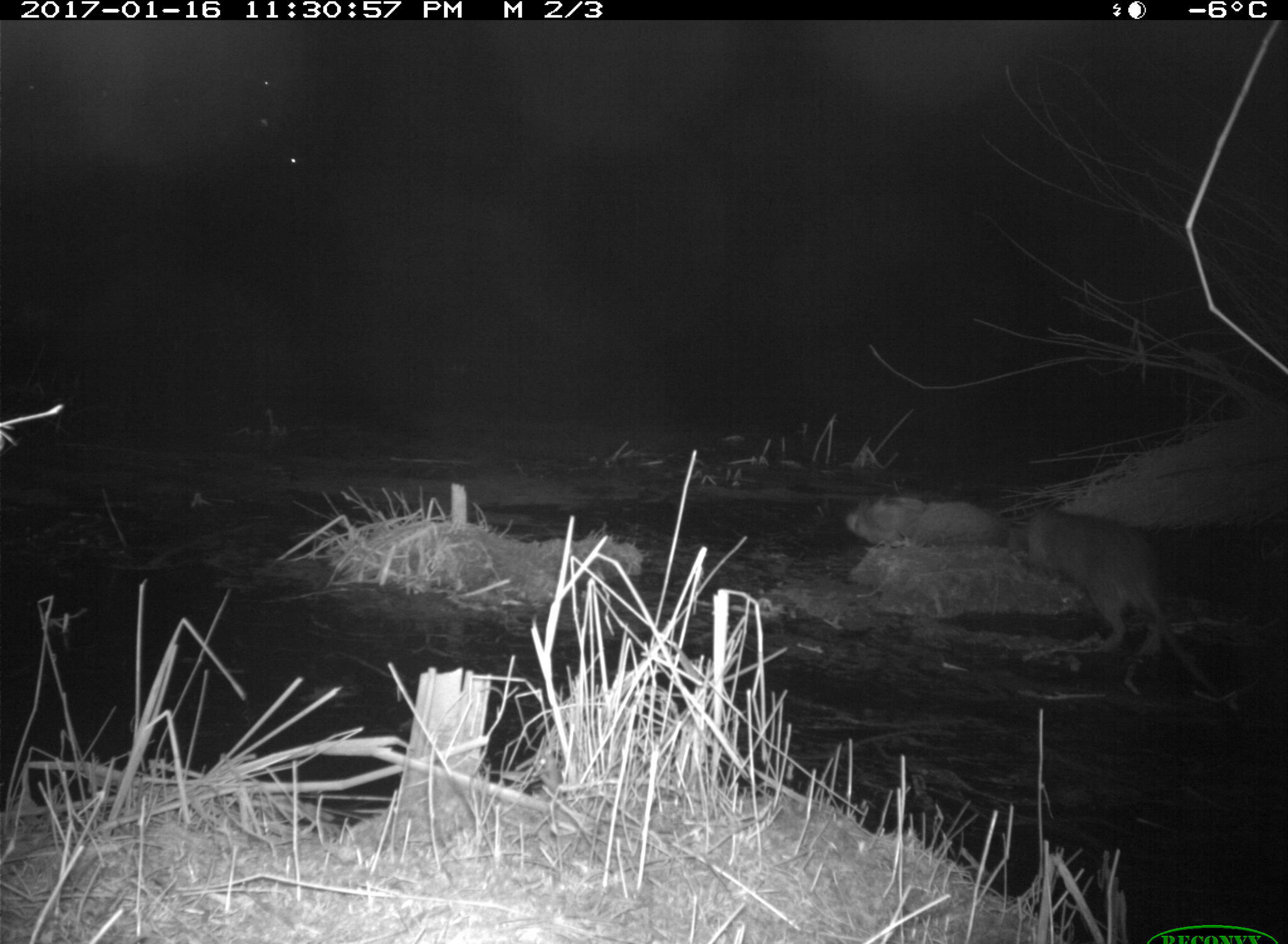

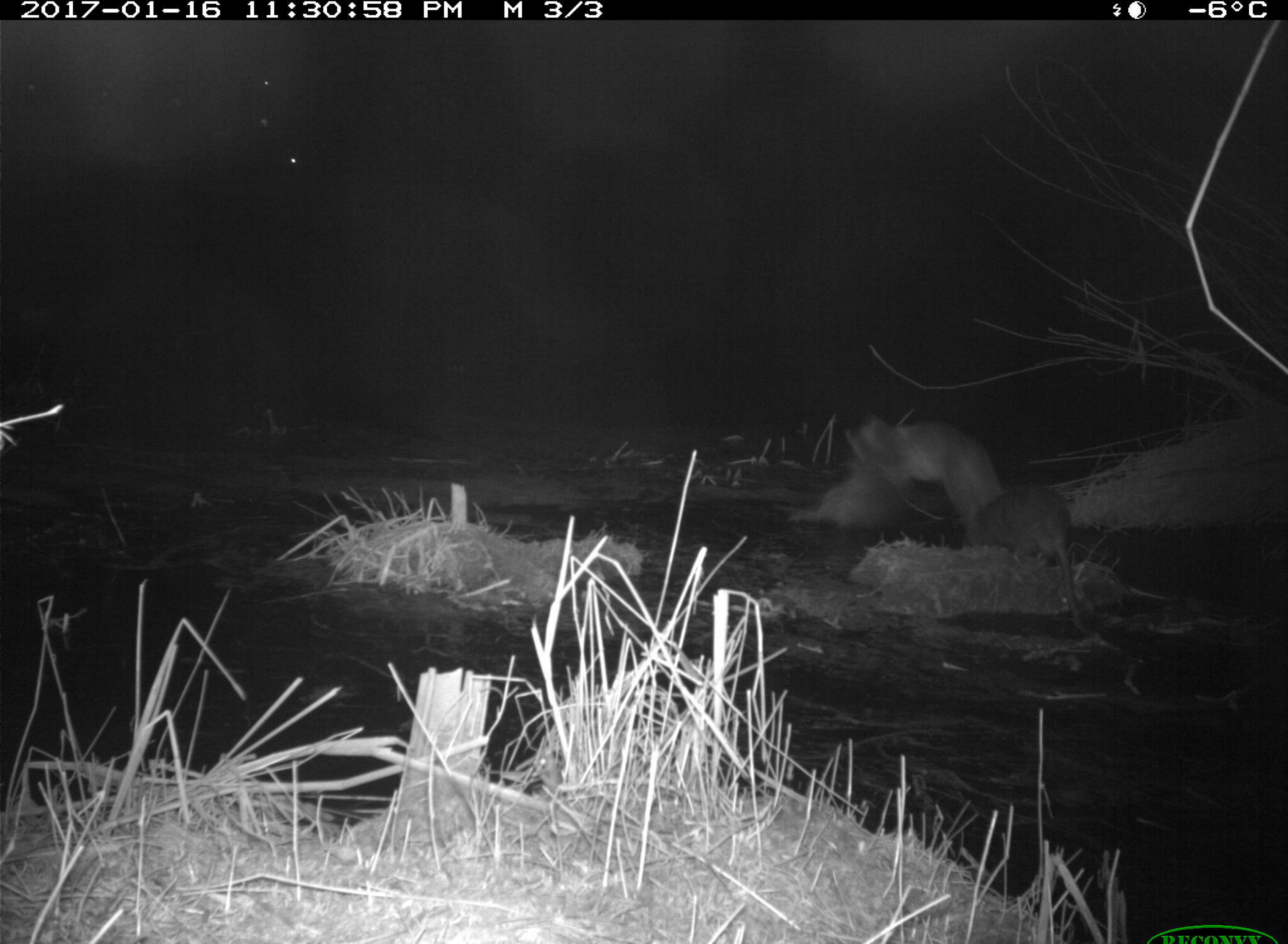

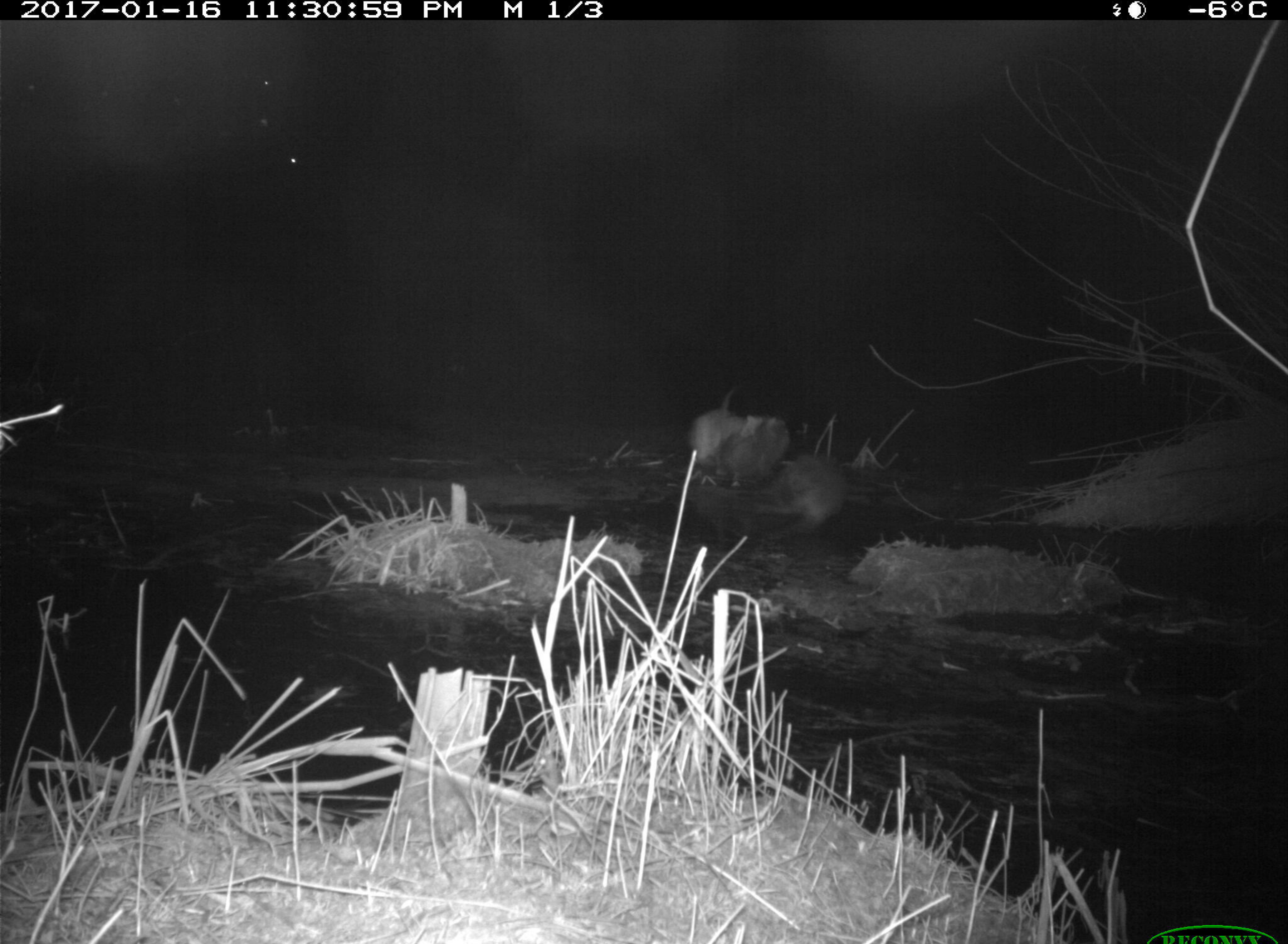

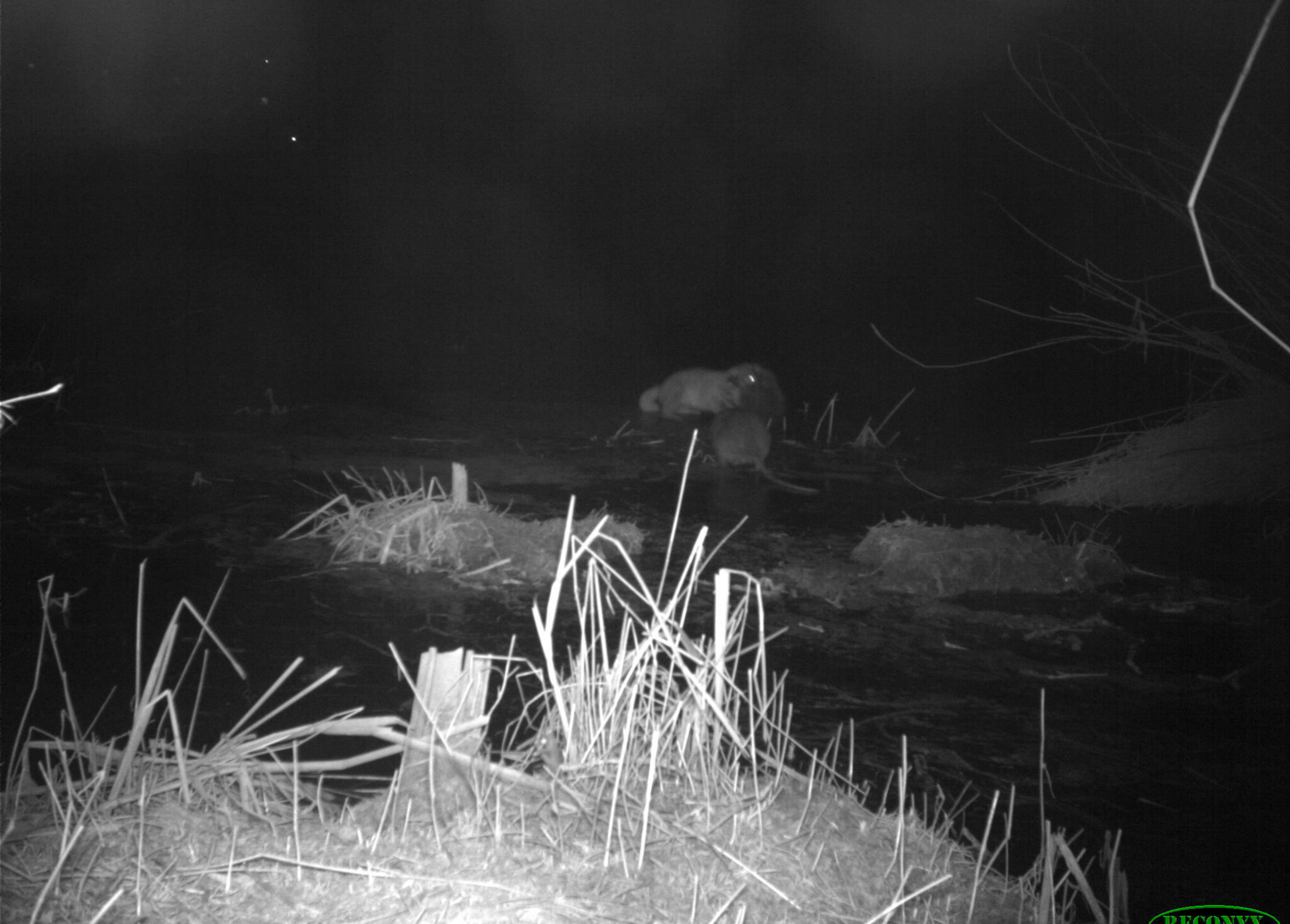

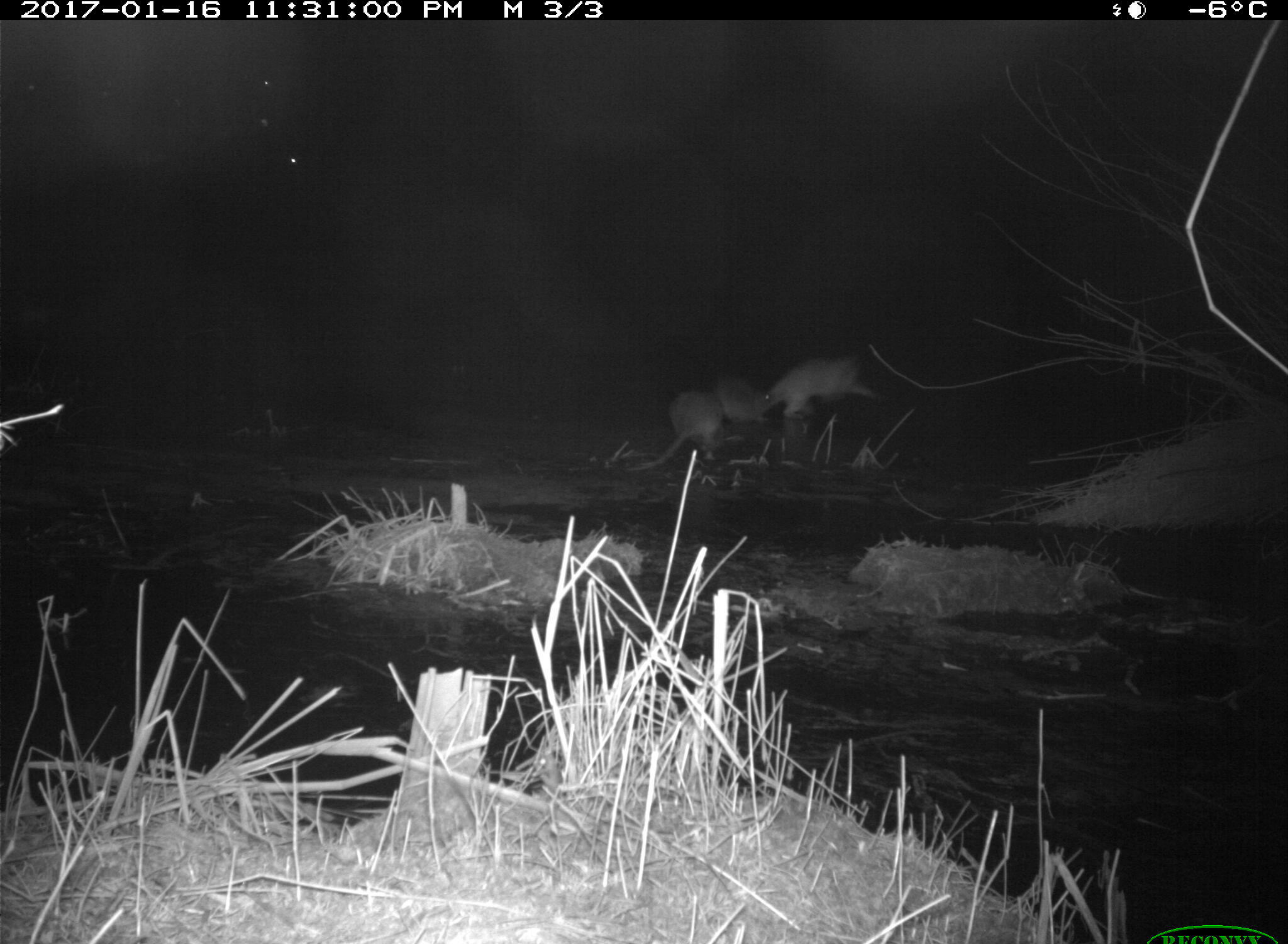

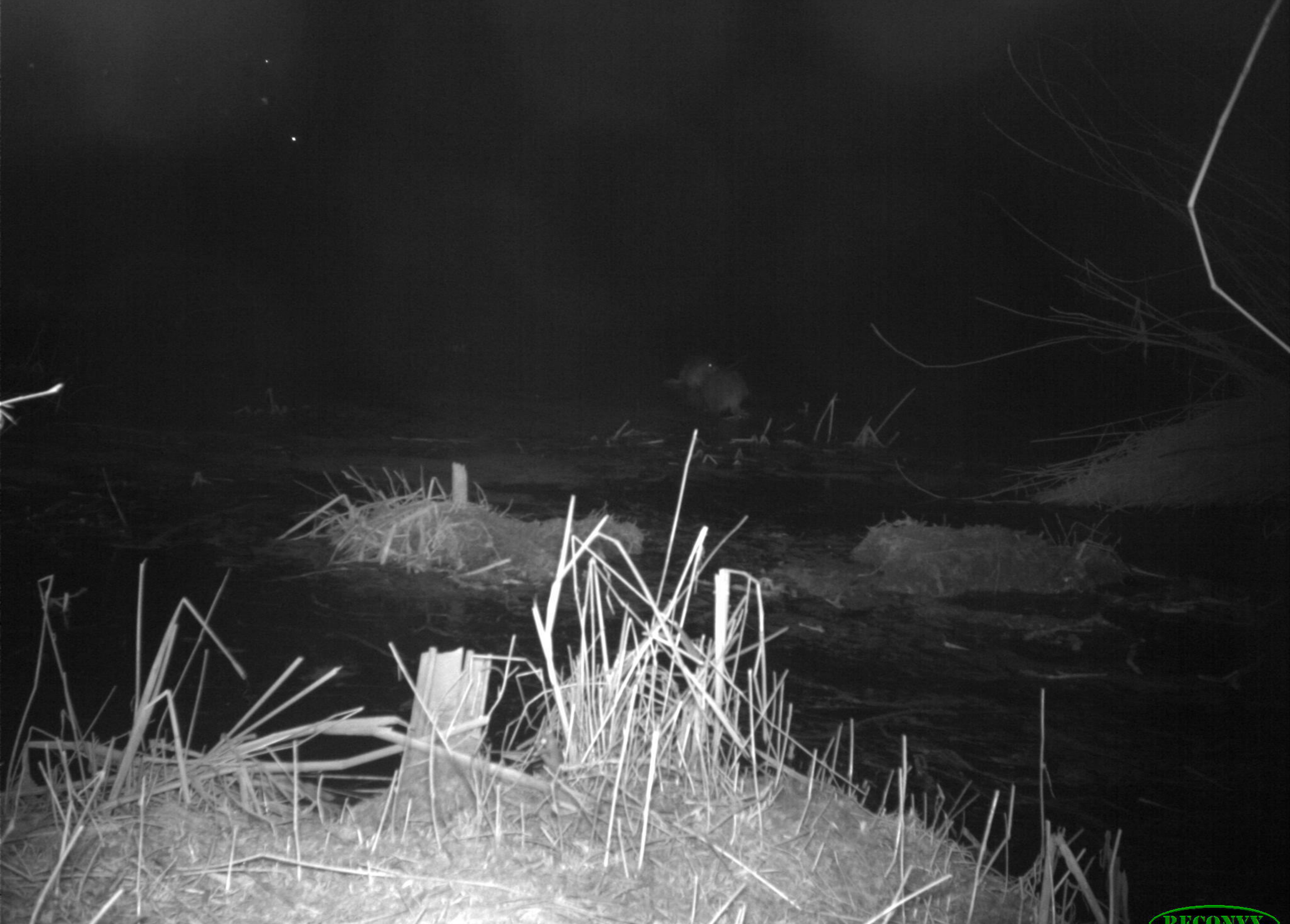

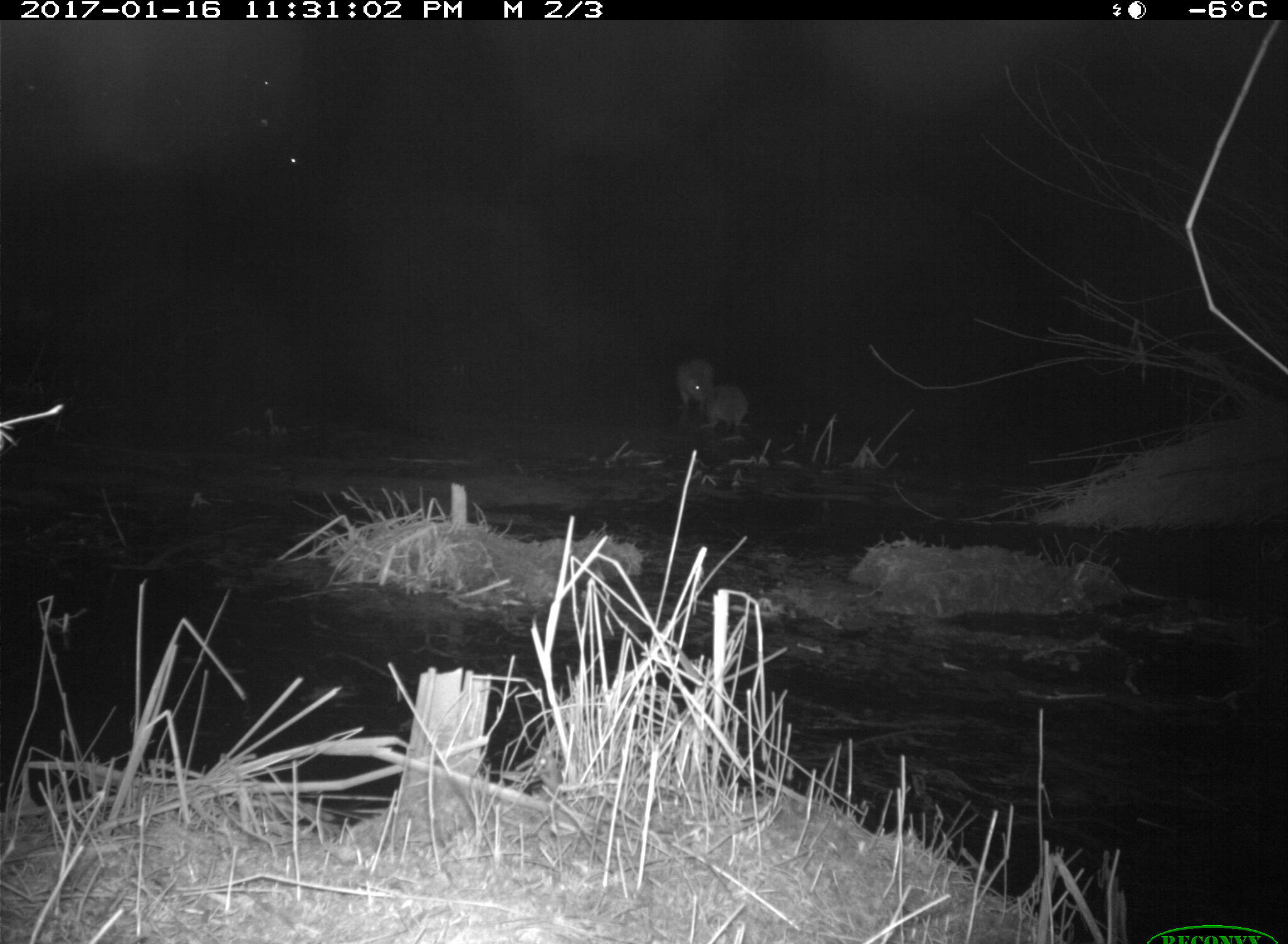

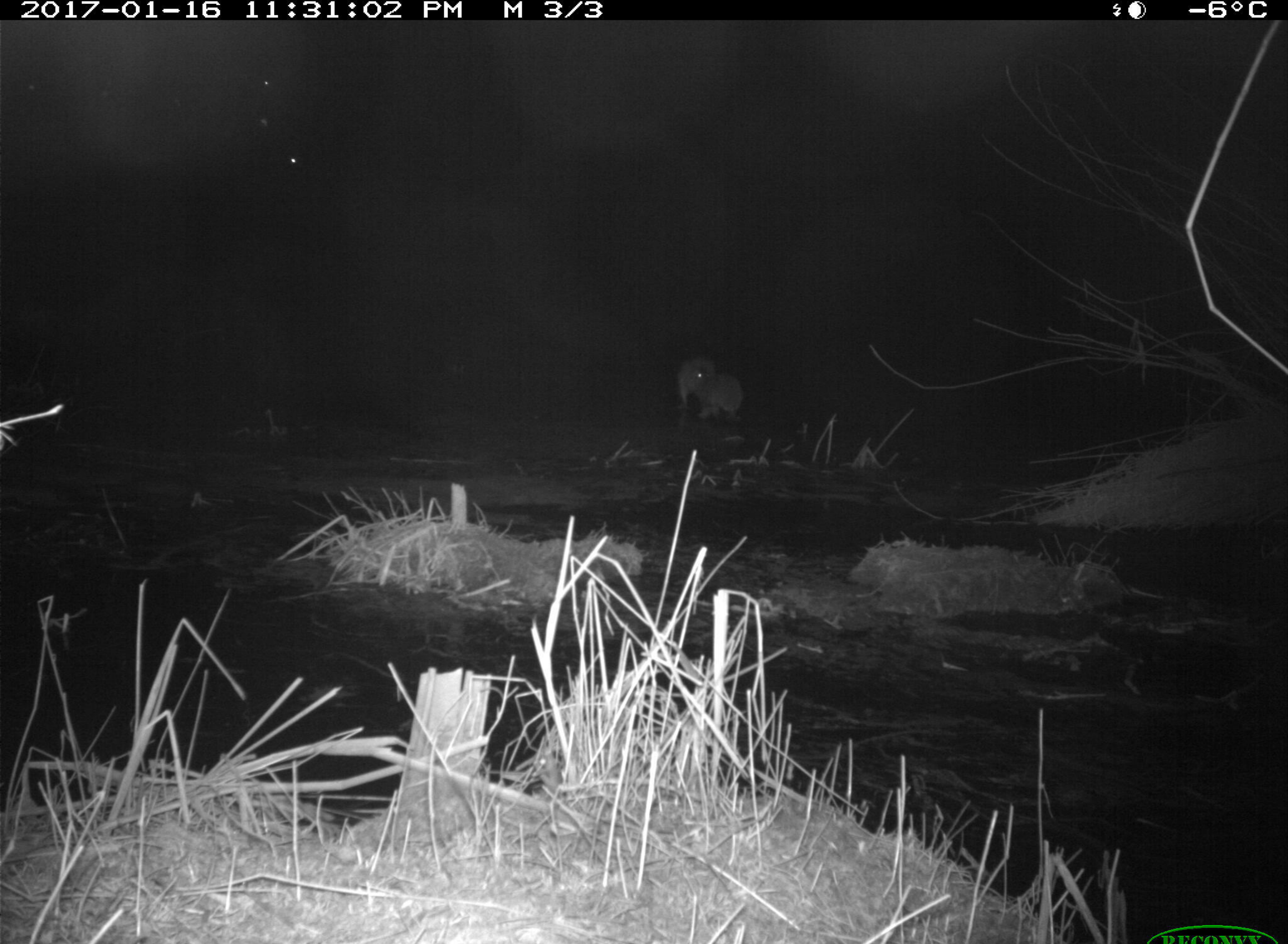

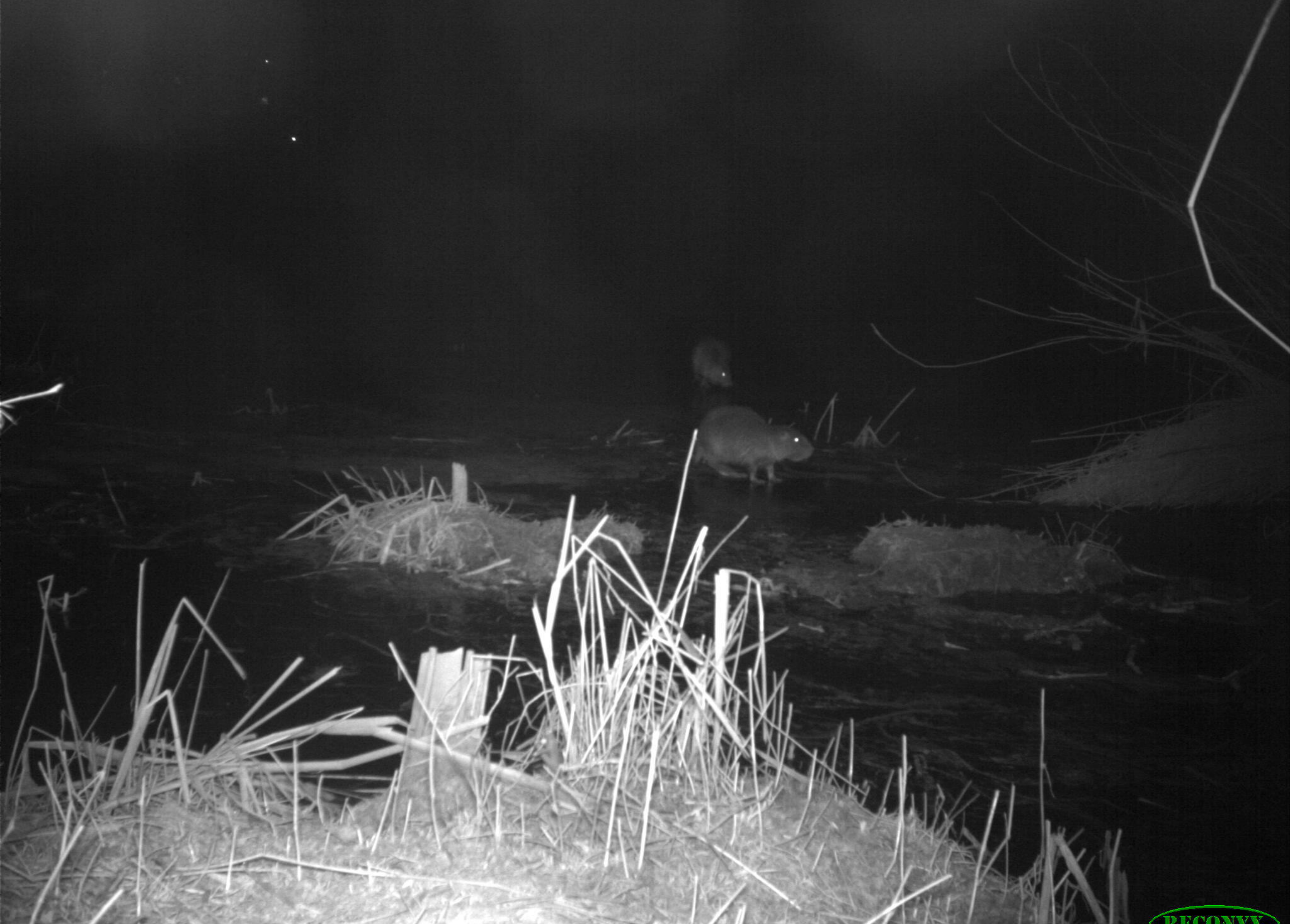

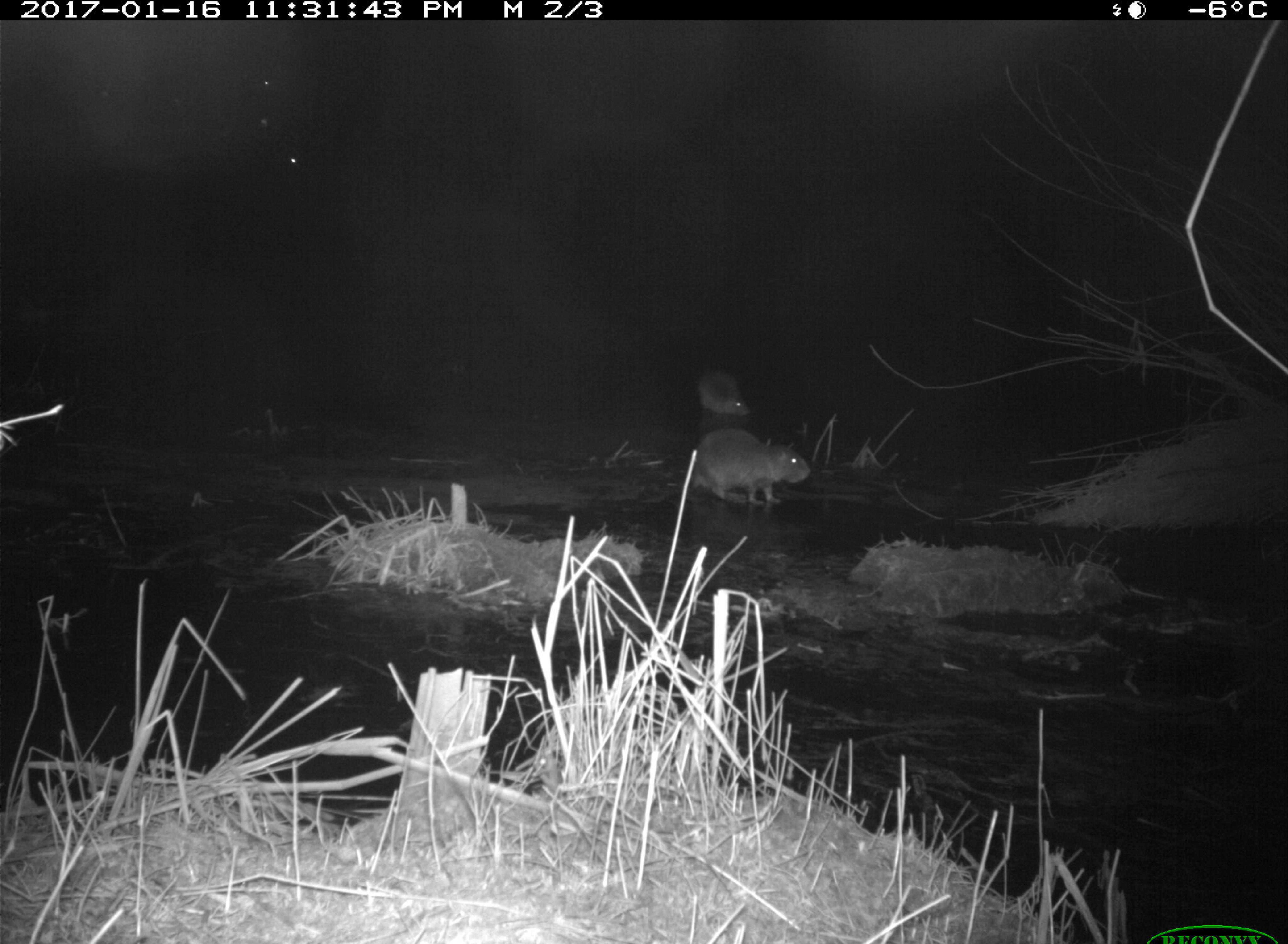

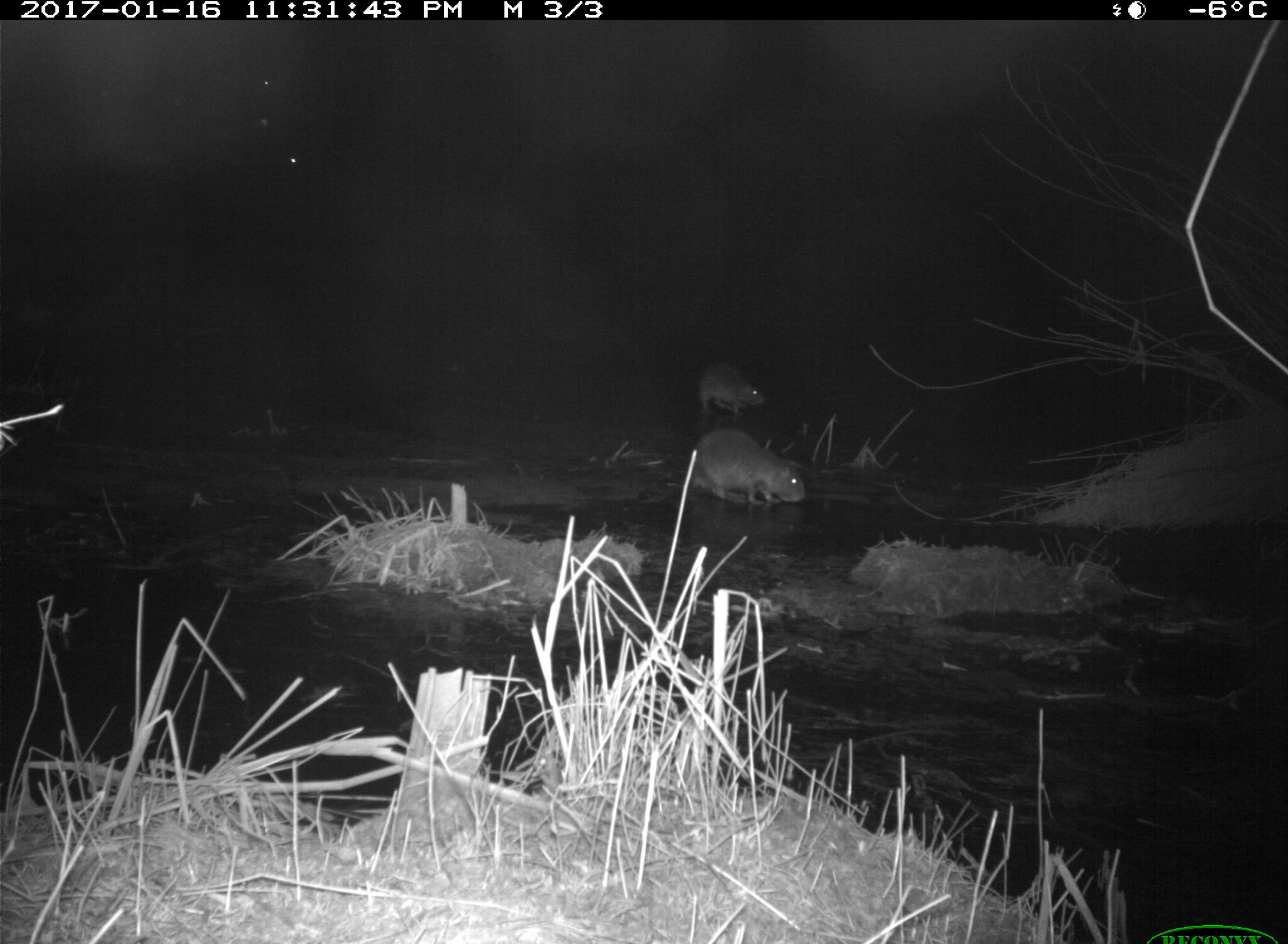

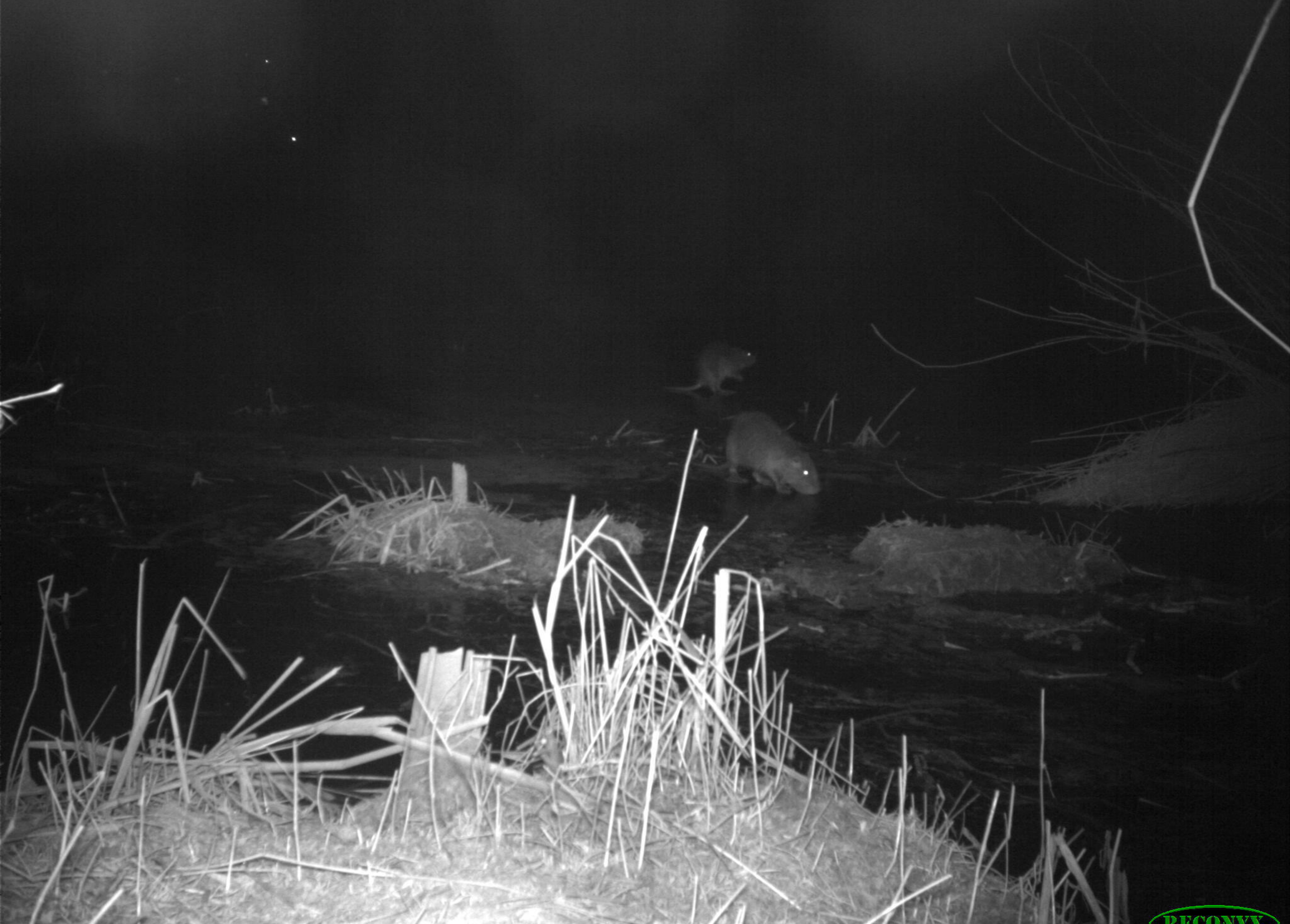

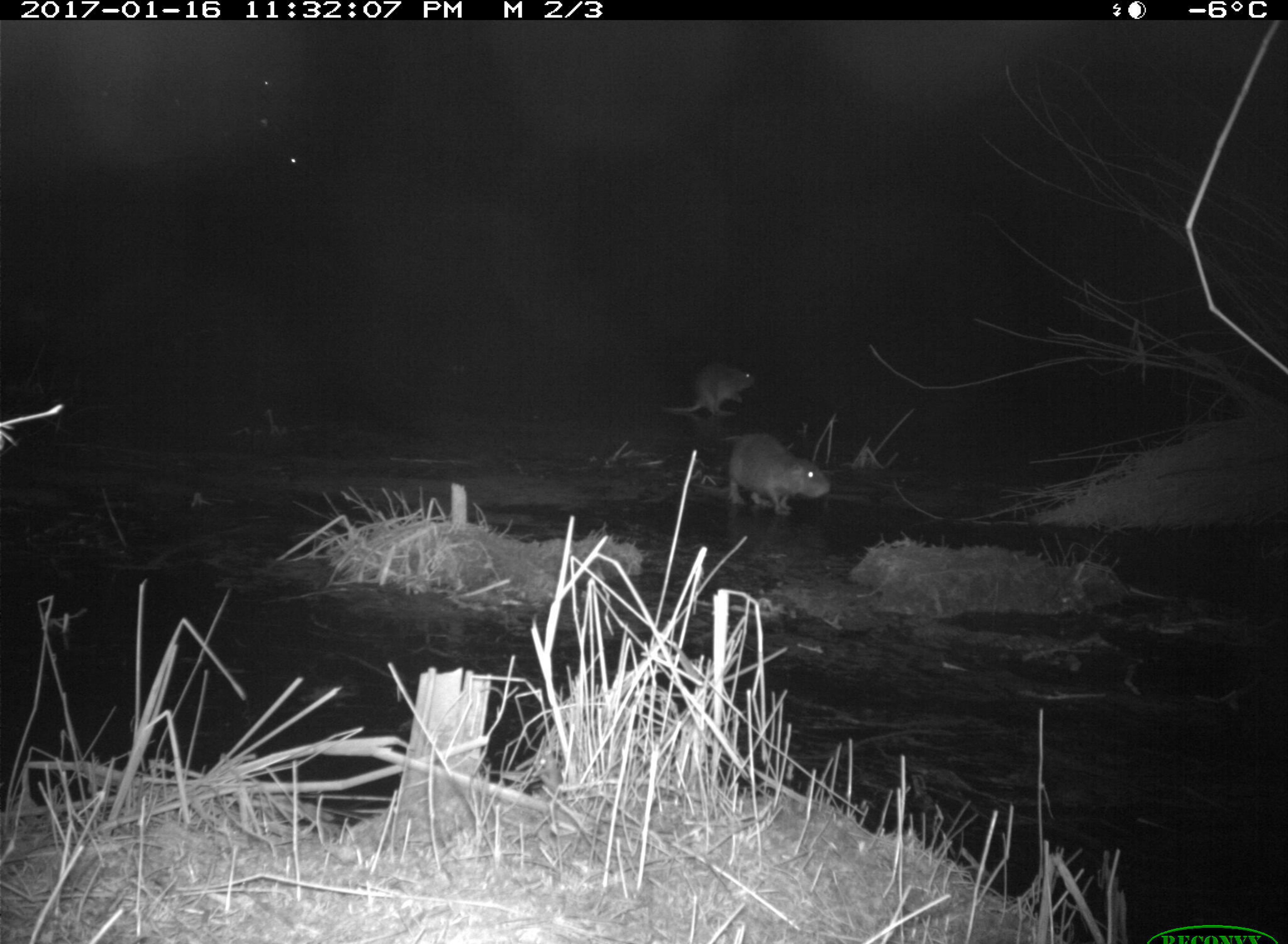

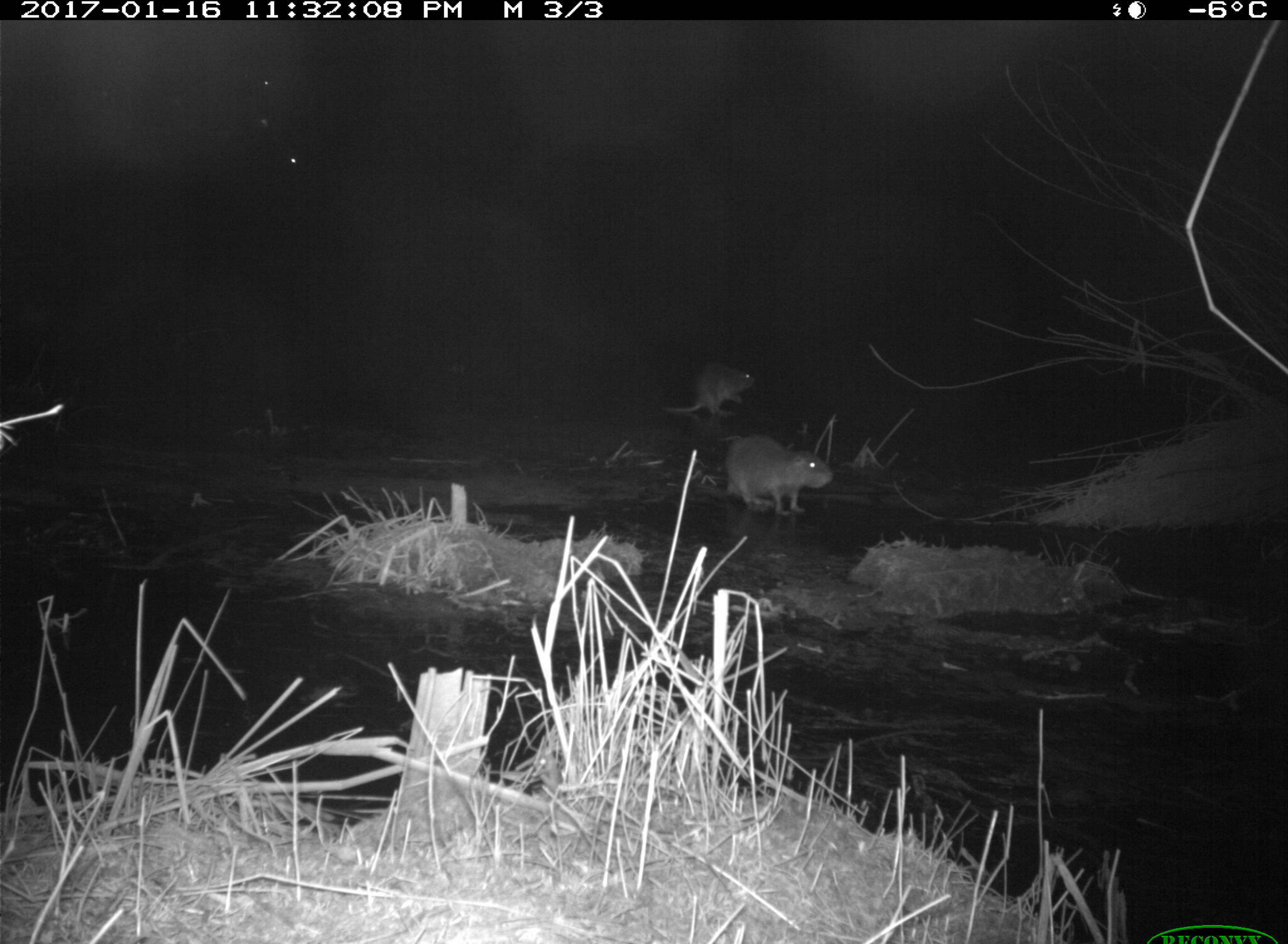

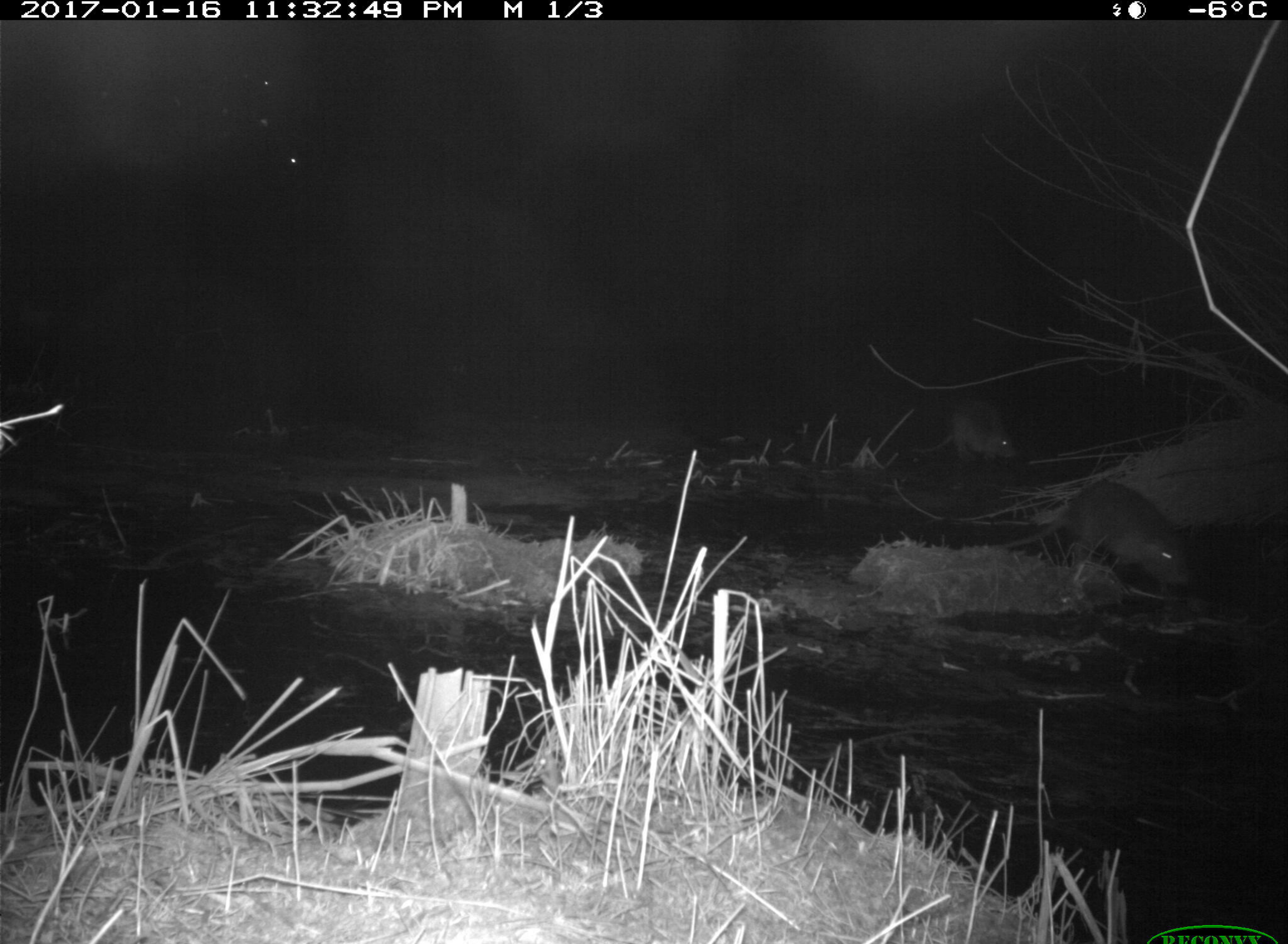

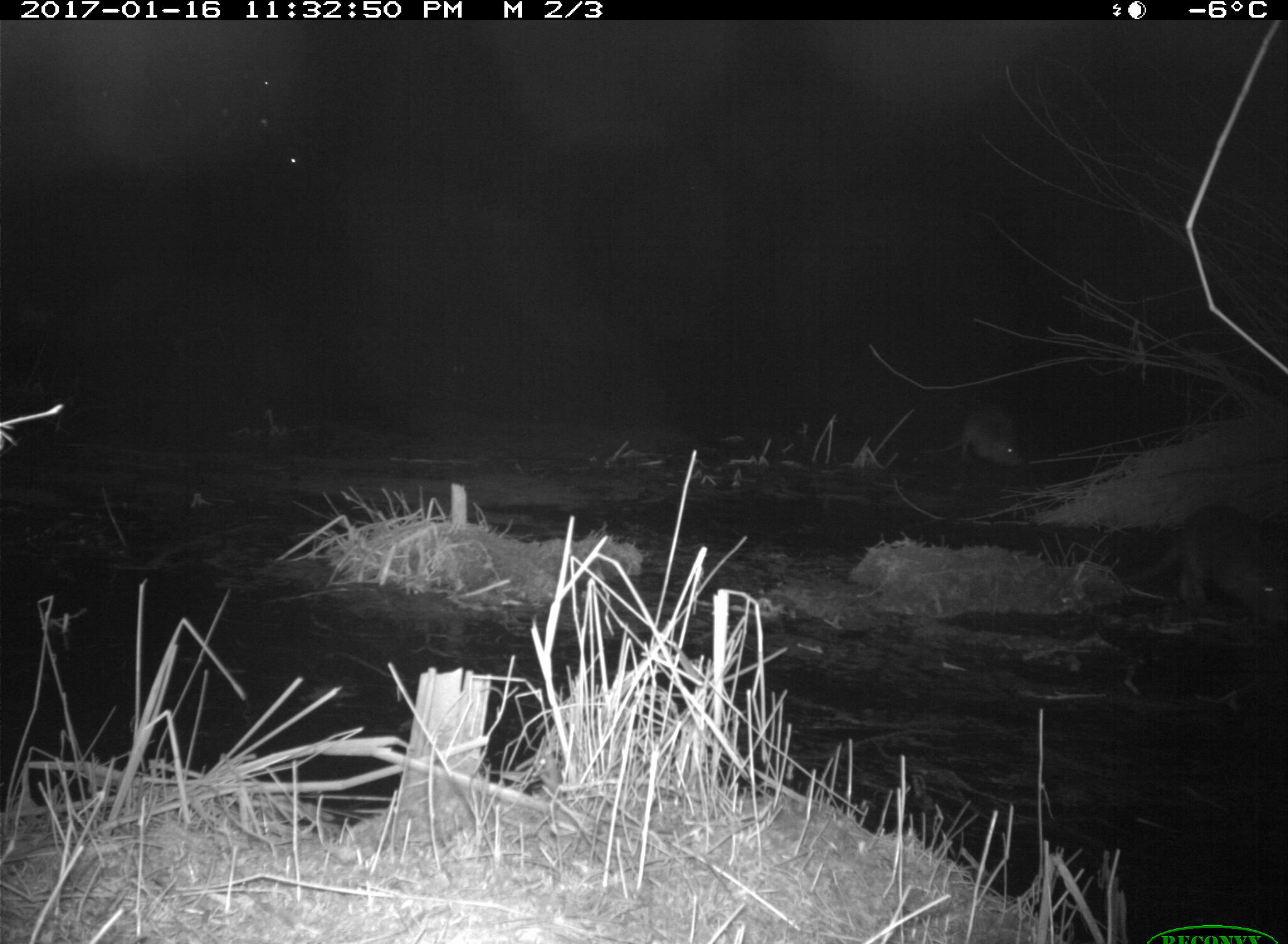

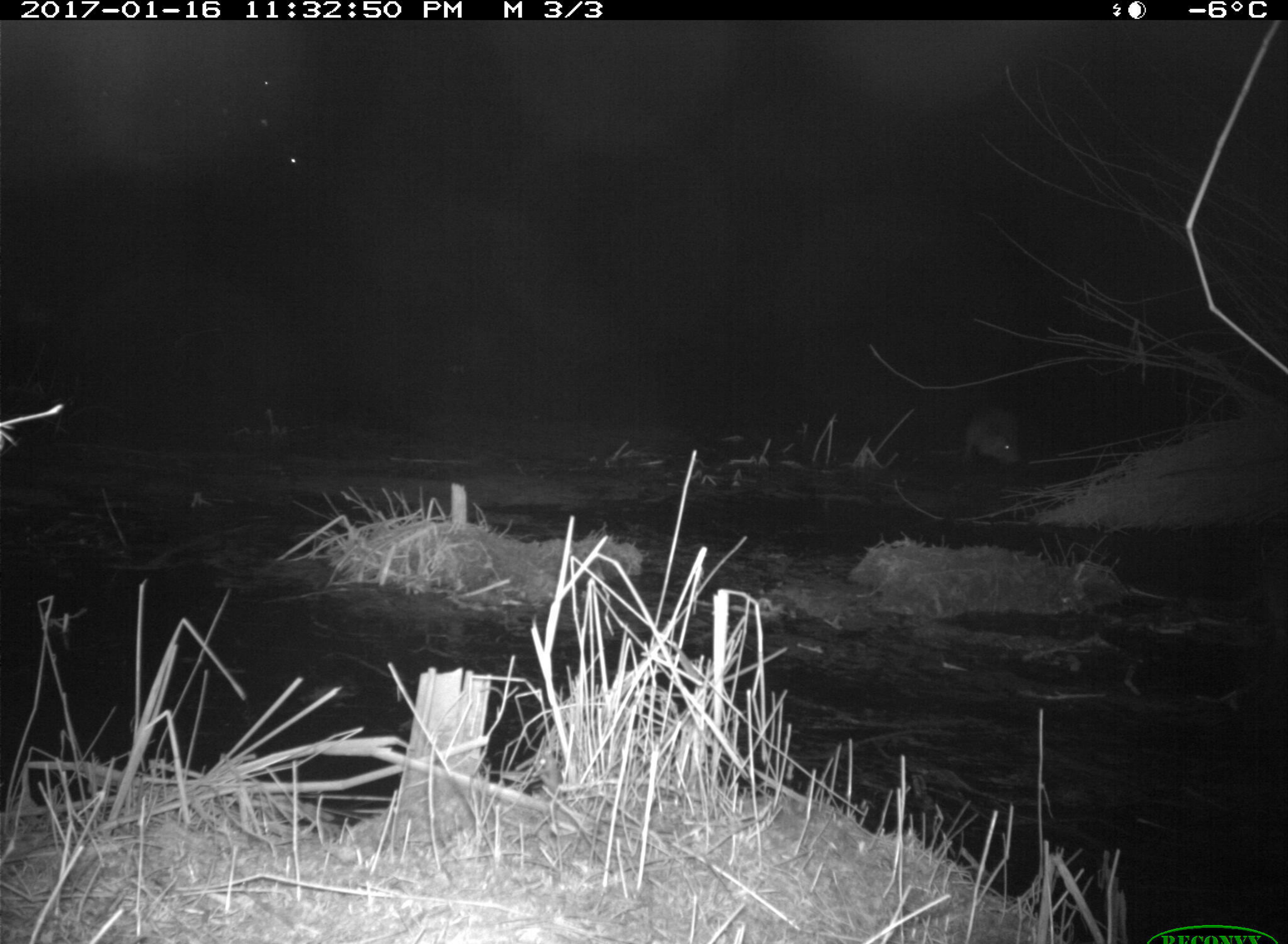

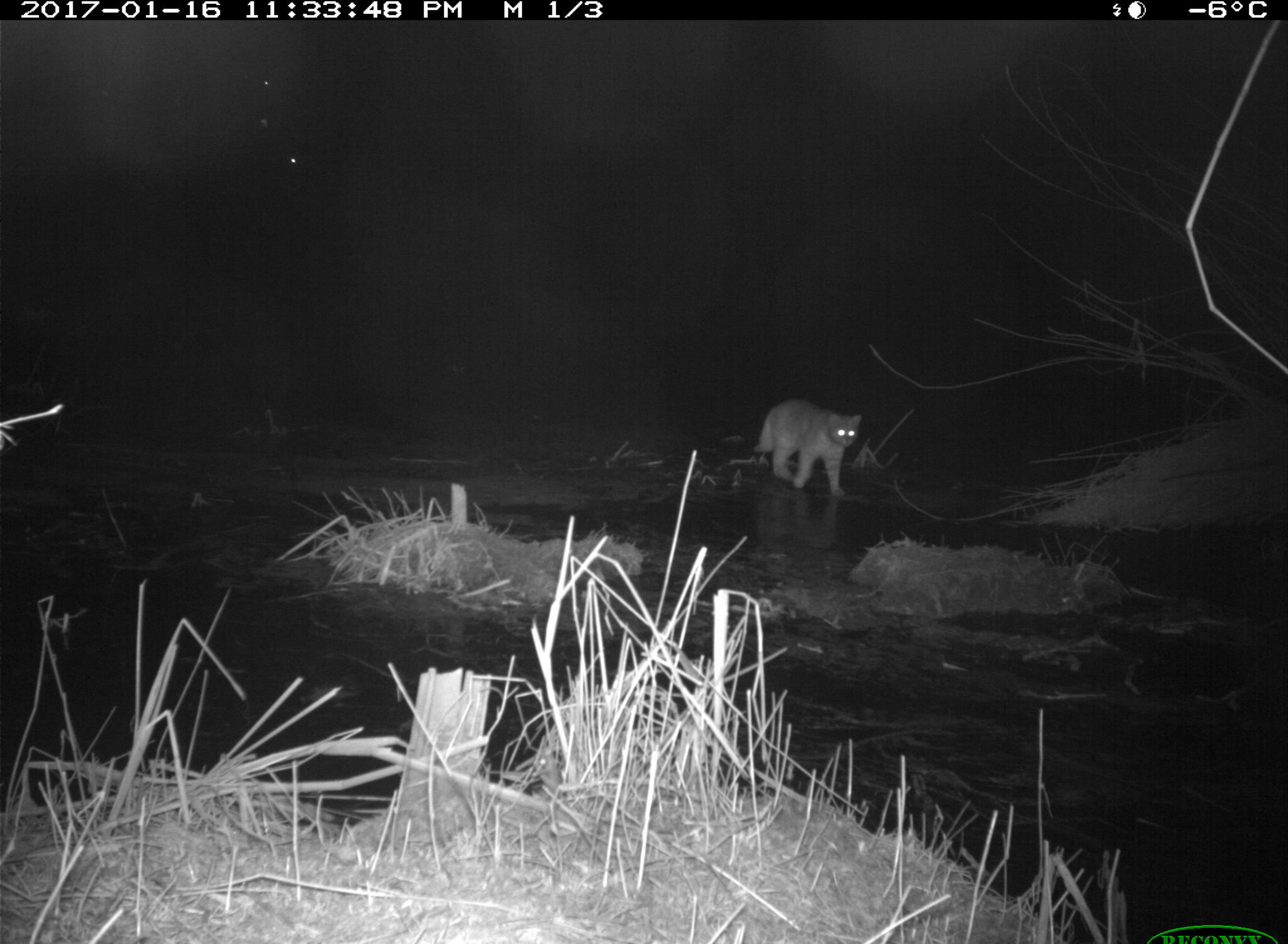

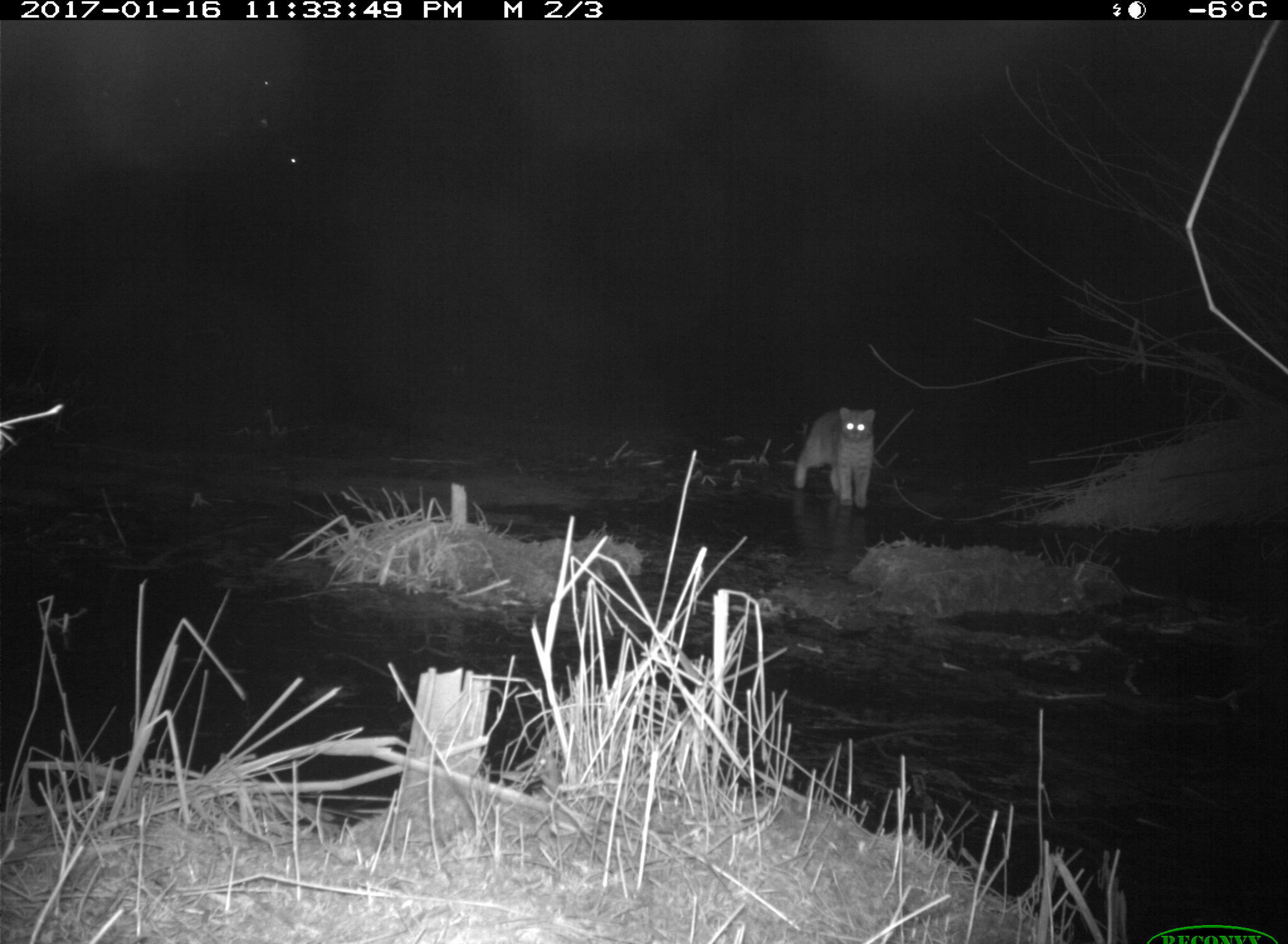

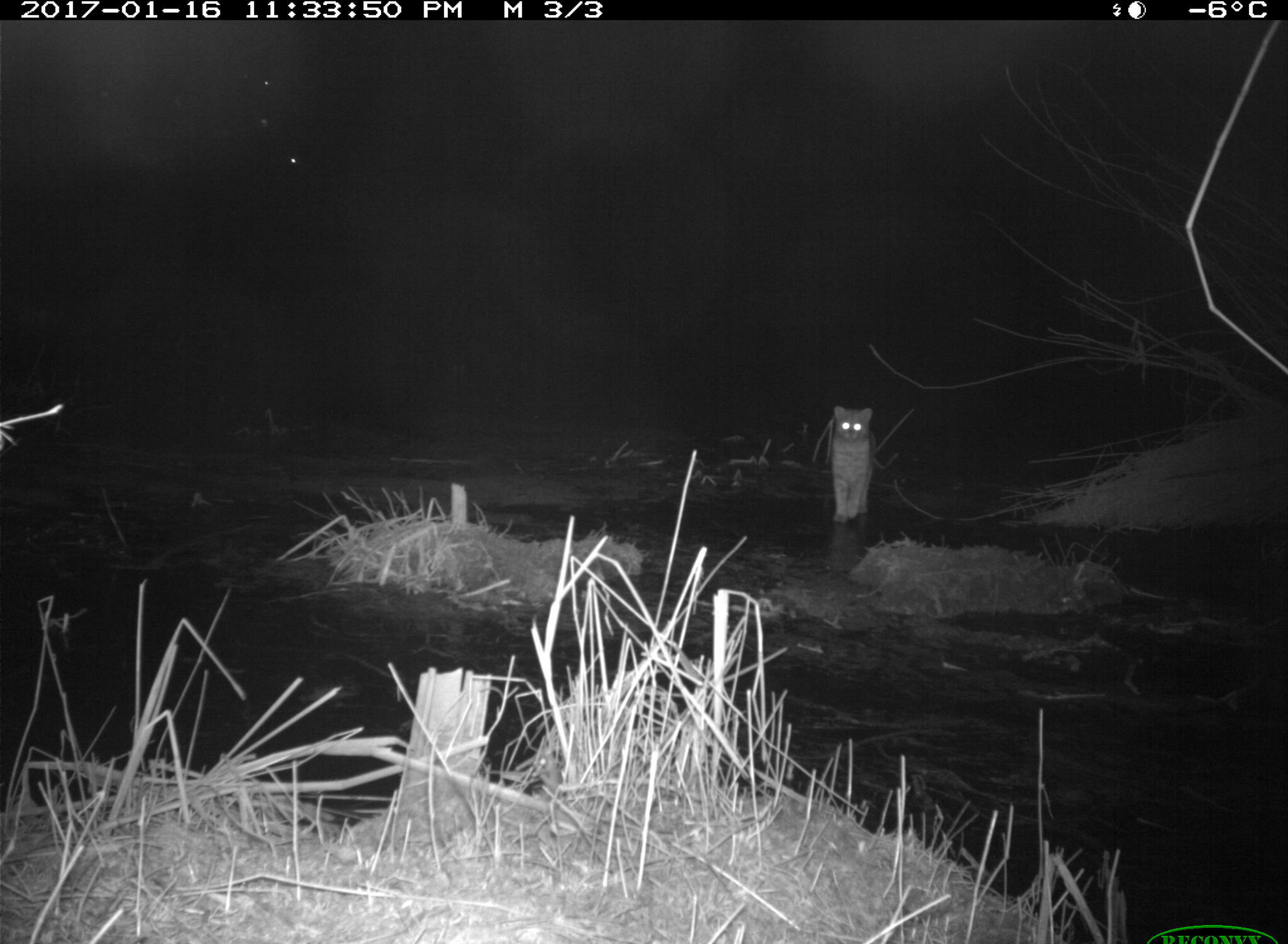

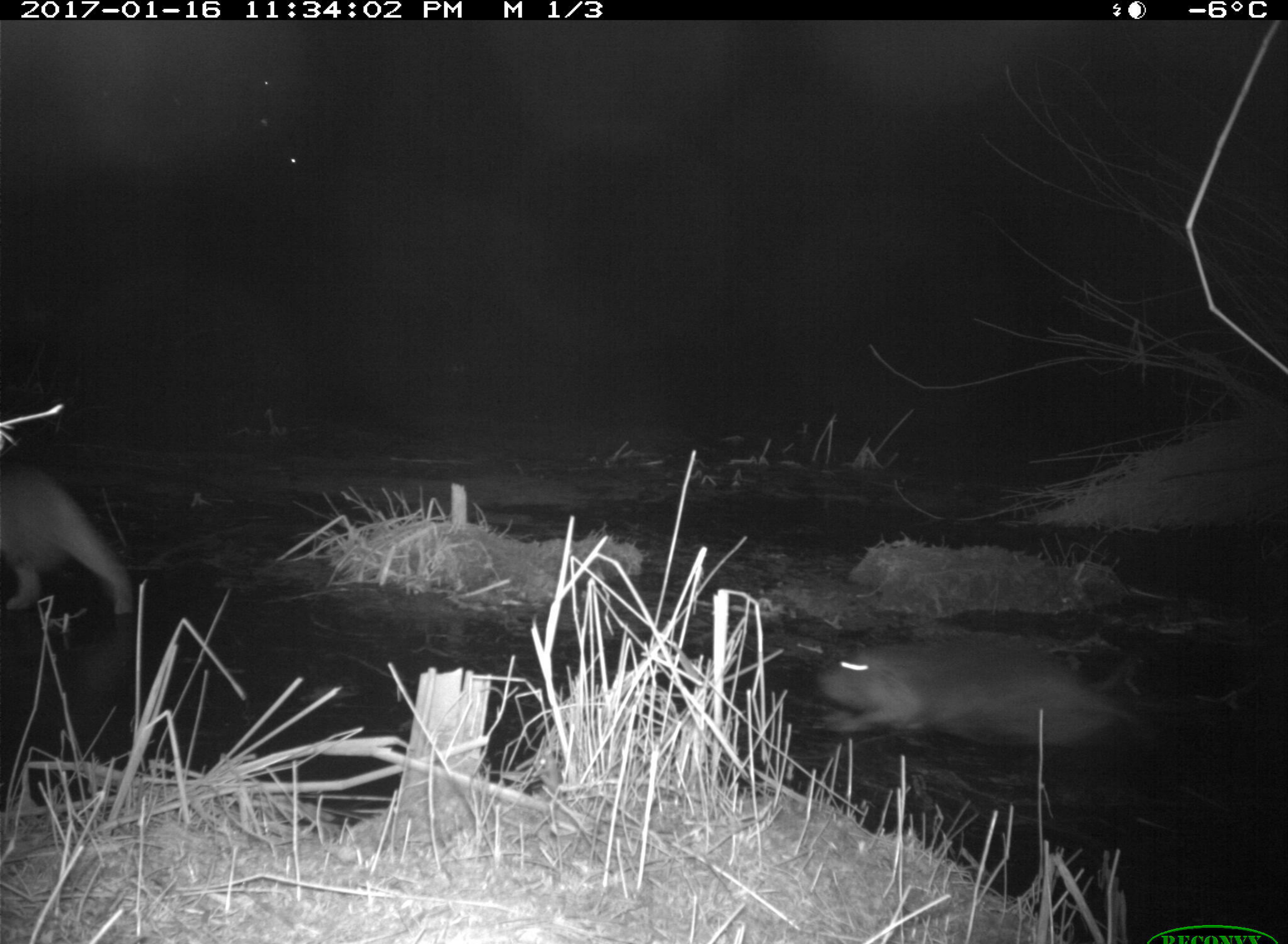

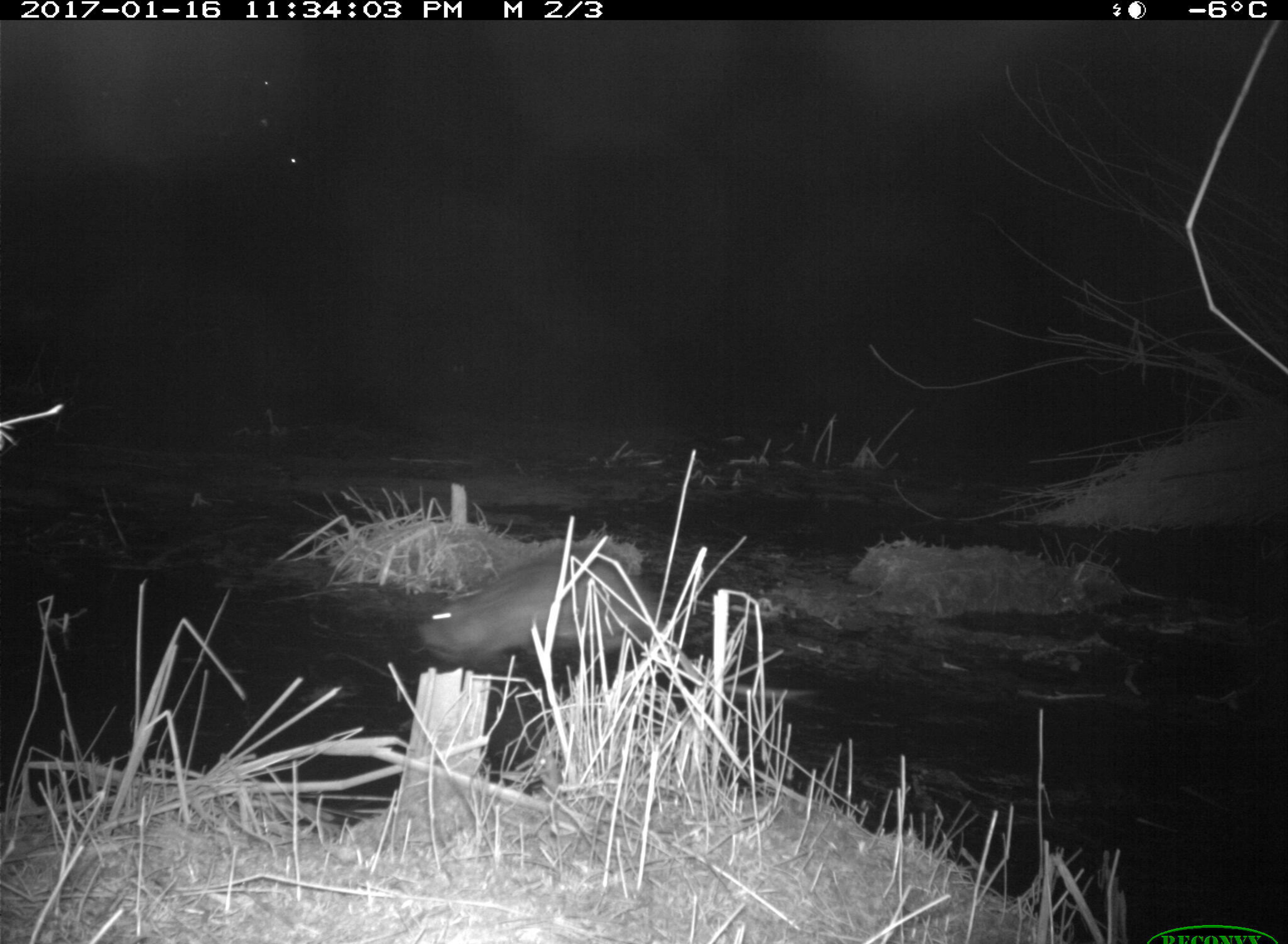

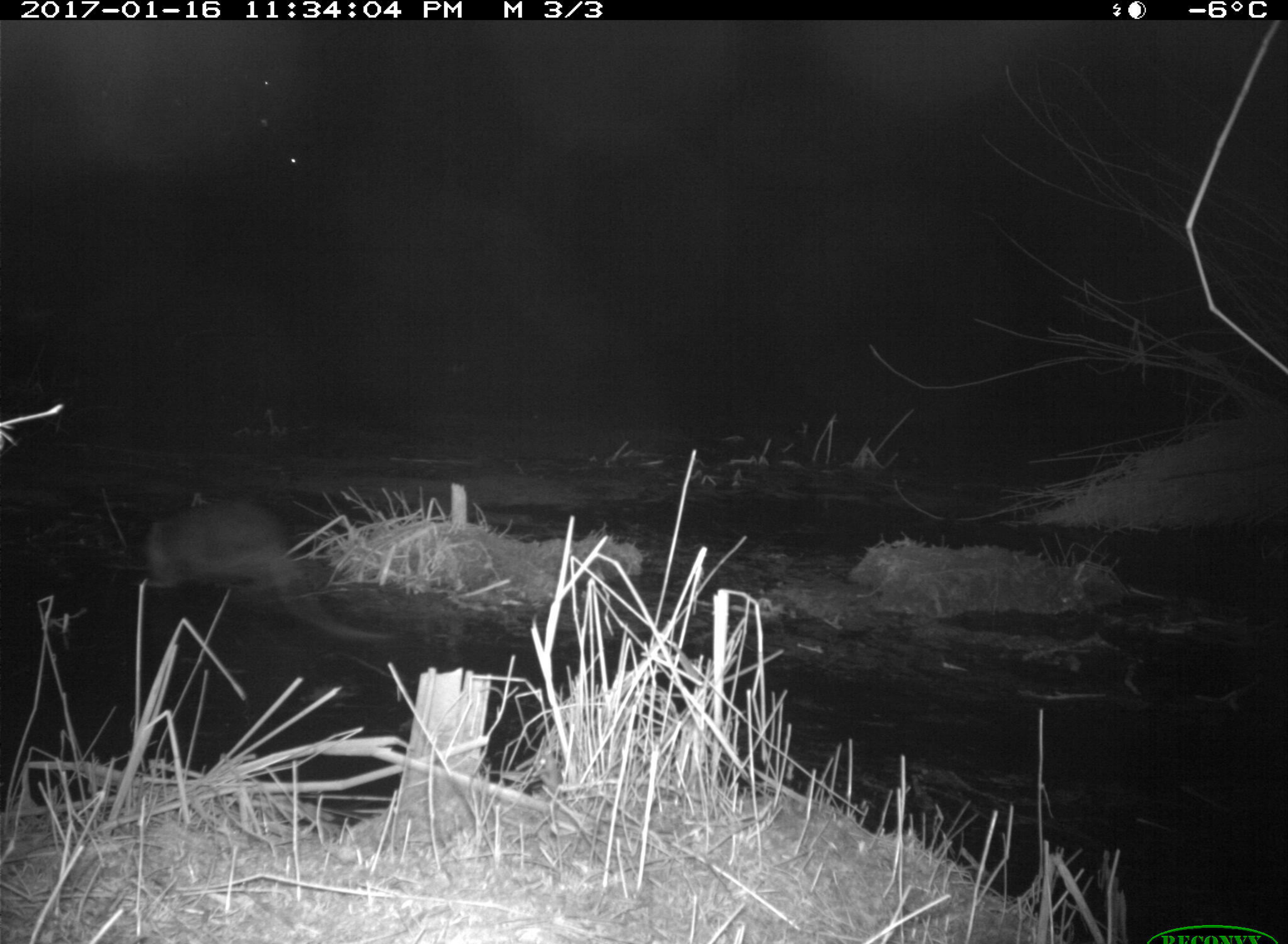

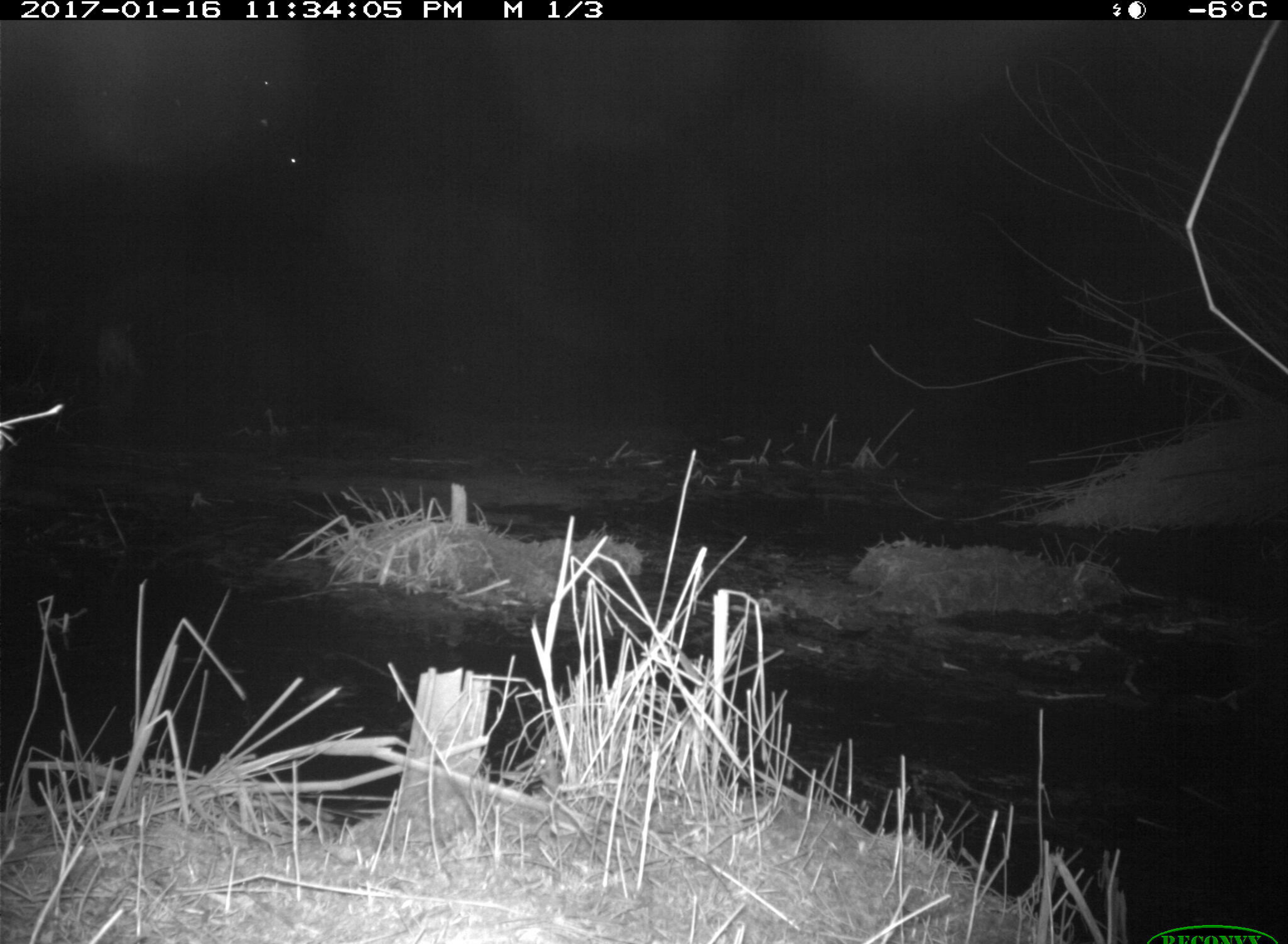

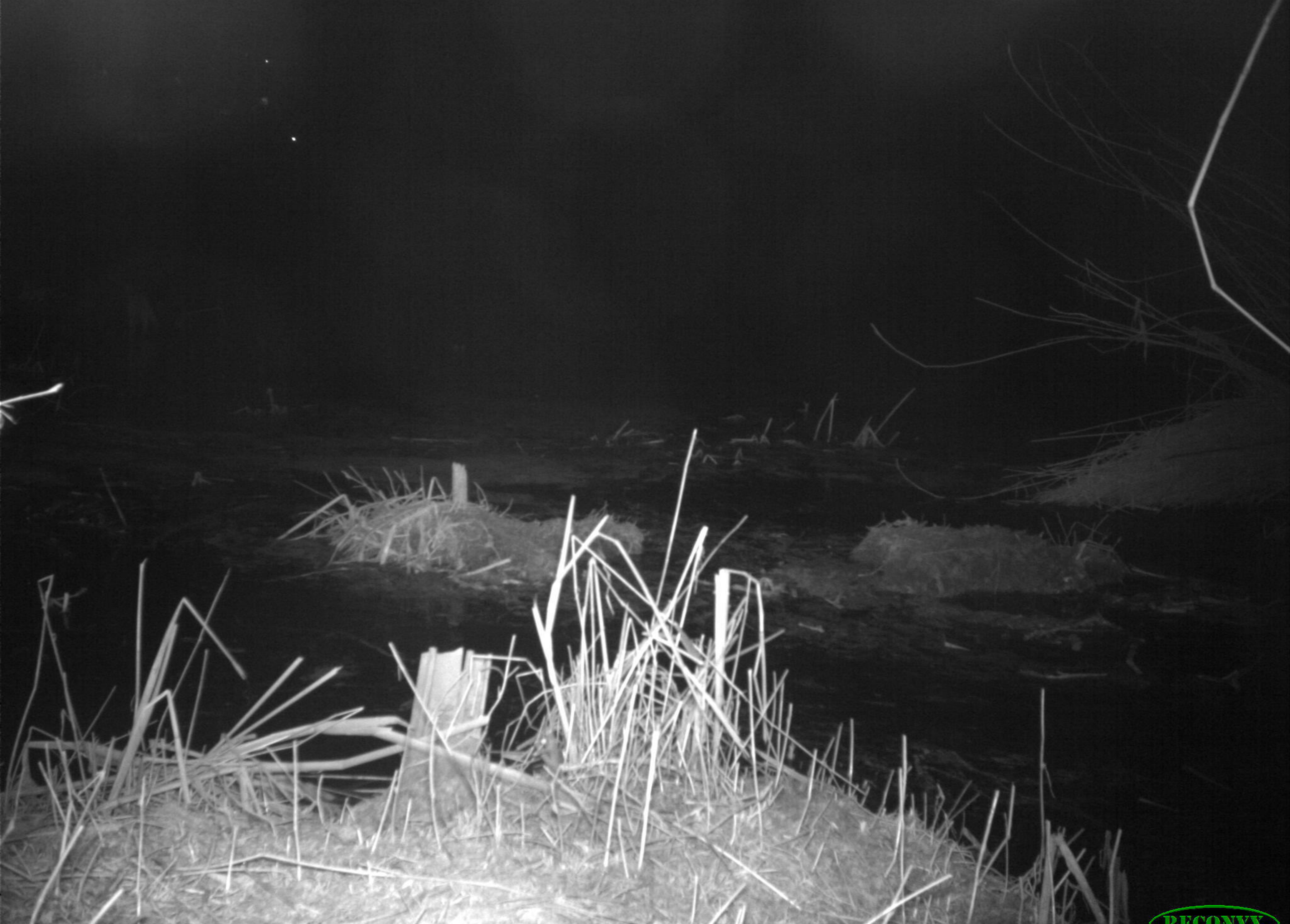

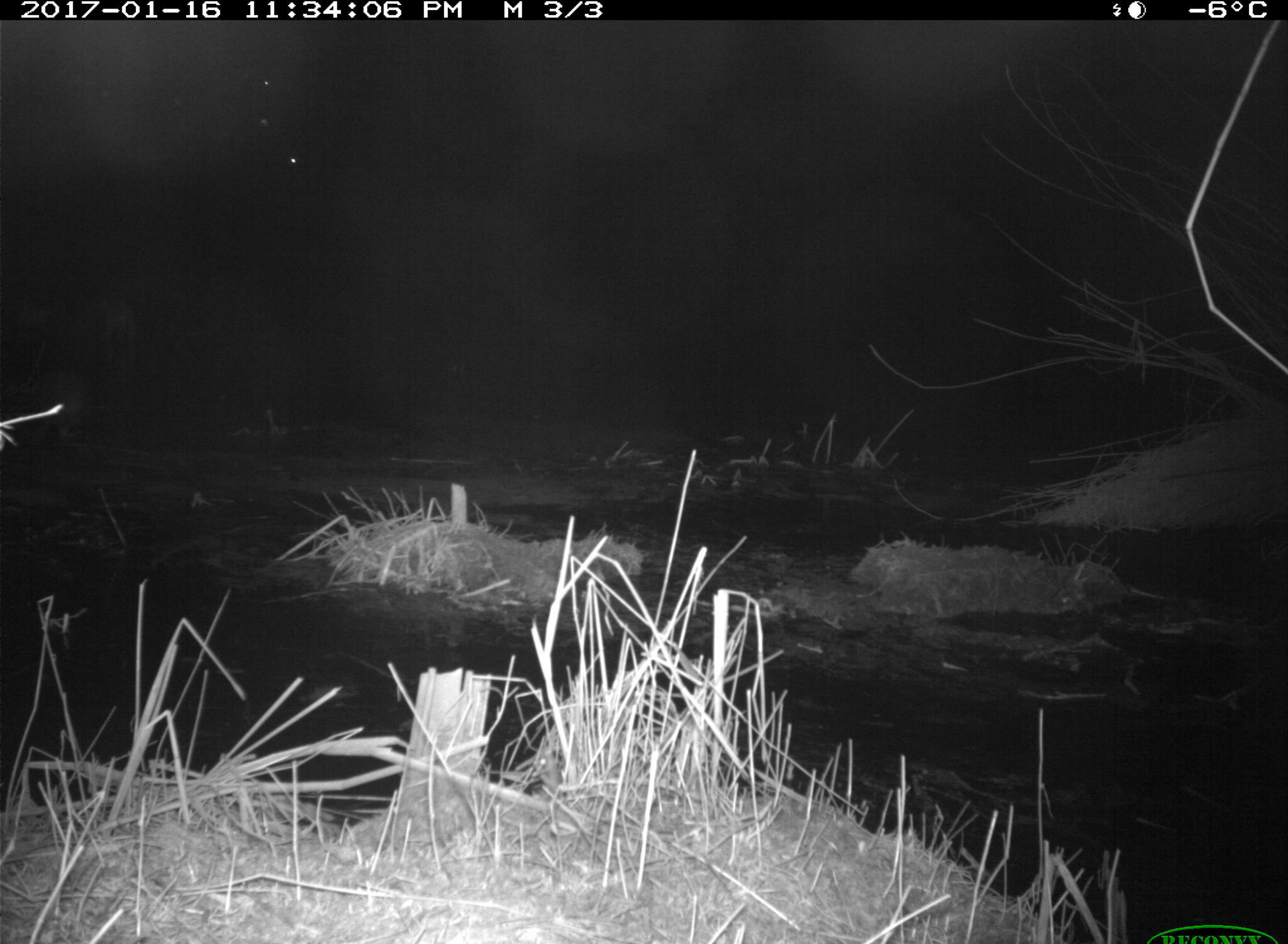

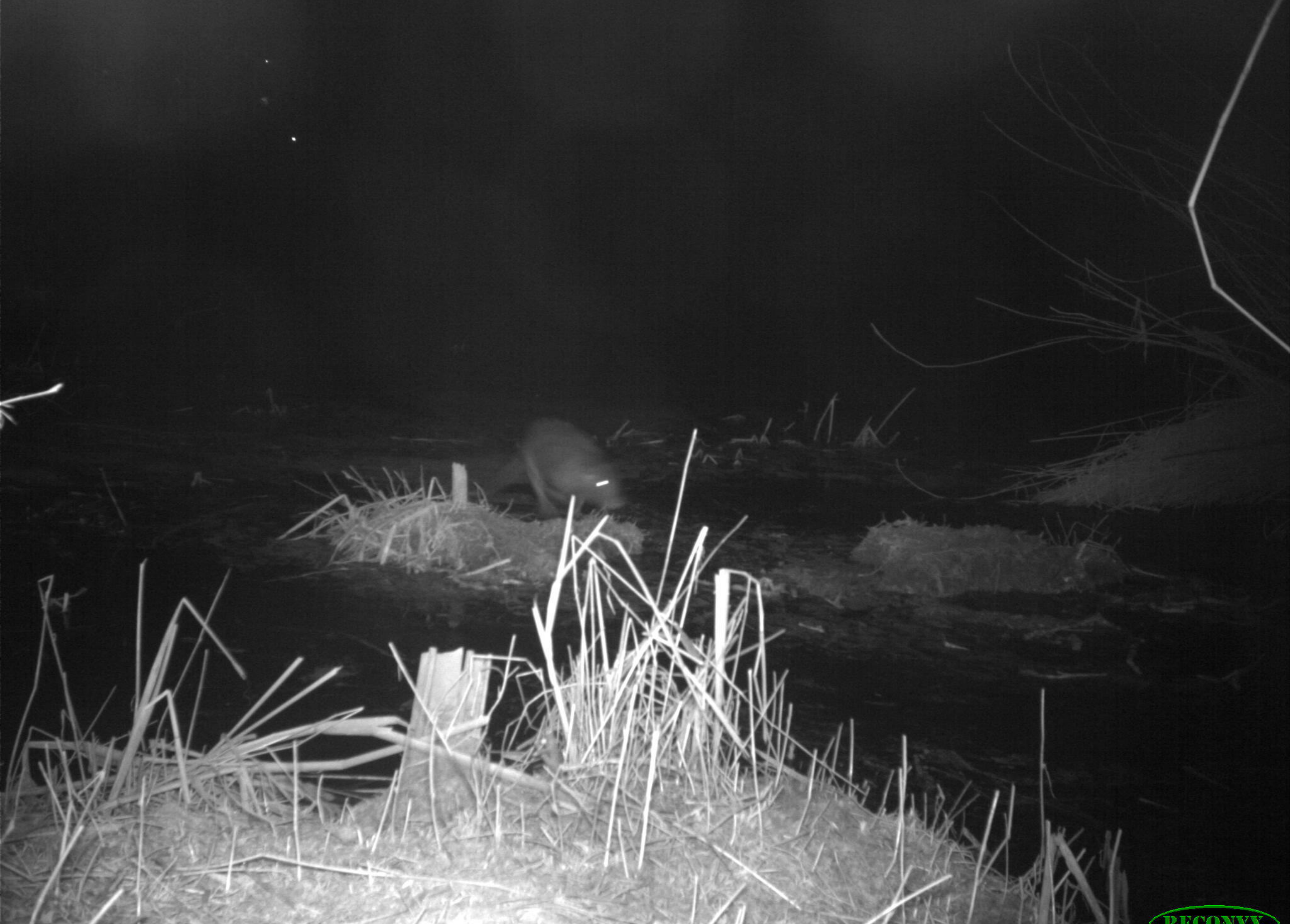

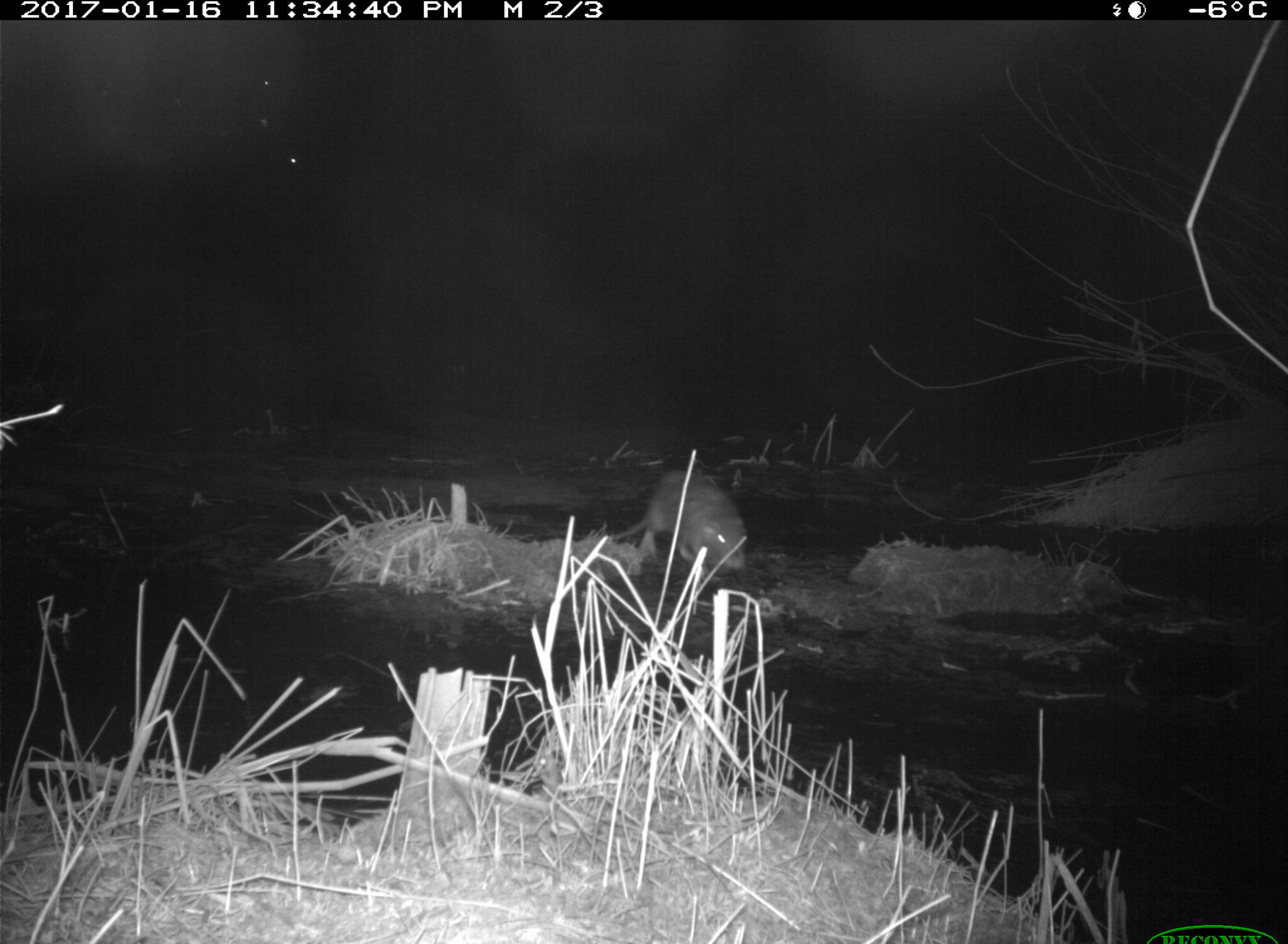

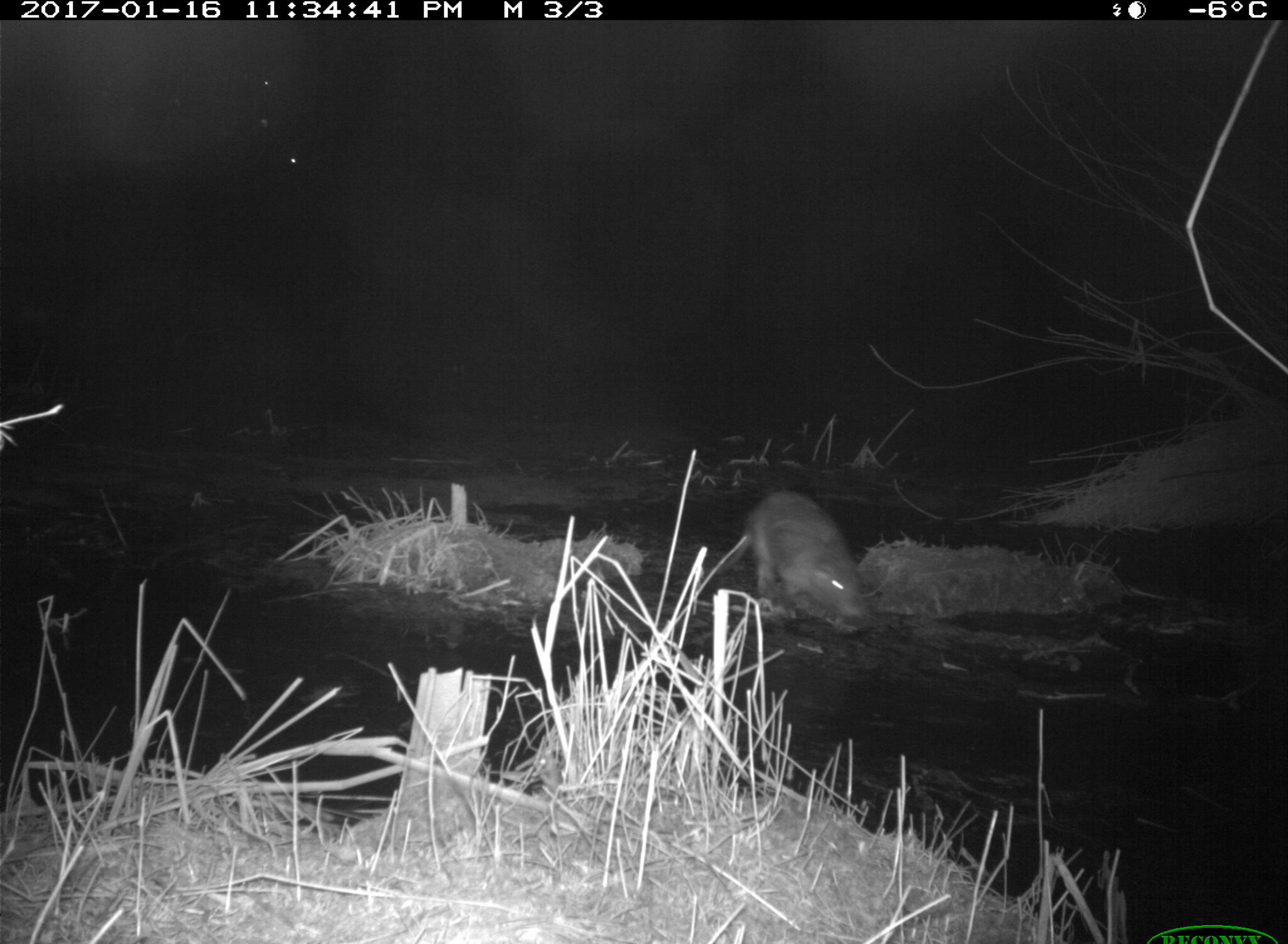

Supplement: Supplementary file 1 — Data S1. [file ECE3-14-e11016-s002.pdf]
