# Supplementary material for: New insights into predator–prey dynamics: First evidence of a leopard cat hunting coypus
Source: Ecol Evol. 2024 Feb 13;14(2):e11016. doi: 10.1002/ece3.11016 (PMC10862177; doi:10.1002/ece3.11016)
Supplement: Supplementary file 2 — Video S1. [file ECE3-14-e11016-s001.zip › Video S1 captions.docx]

Here is the caption for the video S1: 

A video composed of sequential photos capturing predatory and defensive behaviors exhibited by a leopard cat and coypus.
